# Multi-criteria decision analysis of dendrimer applications in drug delivery systems

**DOI:** 10.1140/epje/s10189-026-00633-4

**Published:** 2026-09-24

**Authors:** Zainab Bibi, Muhammad Saad Ghafar, Yasir Ali, Muhammad Imran, Yilun Shang

**Affiliations:** 1 Department of Basic Sciences and Humanities, College of Electrical and Mechanical Engineering, National University of Sciences and Technology, Rawalpindi, 45200 Pakistan; 2 Department of Mathematics Computer Science, National University of Modern Languages, Islamabad, Pakistan; 3 Department of Electrical Engineering, Prince Mohammad Bin Fahd University (PMU), P. O. Box 1664, 31952 Al Khobar, Saudi Arabia; 4 School of Computer Science, Northumbria University, Newcastle upon Tyne, NE1 8ST UK

## Abstract

**Abstract:**

Dendrimers are highly branched, well-defined macromolecules characterized by exceptional structural precision and functional adaptability. These properties render dendrimers valuable in nanotechnology, drug delivery, and catalysis. However, selecting the most suitable dendrimer for drug delivery is difficult because its branching pattern, structural, and physicochemical properties are closely related. In this research, we utilized the multi-criteria decision-making (MCDM) technique to identify potential candidates for drug delivery applications among the four considered families: phosphorus-containing, PDI-cored, Triazine-based, and Aliphatic polyamide dendrimers. Both qualitative and quantitative molecular descriptors are integrated within the model. Degree-based topological indices, including the Narumi Katayama (NK), Modified Narumi Katayama (NK$^*$), Forgotten Index (F-index) and Augmented Zagreb Index (AZI) indices, are utilized to capture variations in molecular complexity, stability, and branching efficiency. The evaluation was conducted using entropy-based weights obtained from representative benzene derivatives; also, the equal weighting and sensitivity analyses were utilized to validate the ranking results. This study uses multi-objective optimization on the basis of ratio analysis (MOORA), simple additive weighting (SAW), and the technique for order preference by similarity to ideal solution (TOPSIS) techniques for criteria weighting, ranking, and reflects how topological descriptors support decision-making in dendrimer selection. The results of these investigations provide quantitative guidance for chemists and pharmaceutical researchers on the forecasting capacity of topological indices in designing dendrimers with enhanced drug delivery.

**Graphical abstract:**

Dendrimer structures, graph construction, multi-criteria decision-making, sensitivity analysis, property modeling, and interpretation for dendrimer applications.

**Figure:**
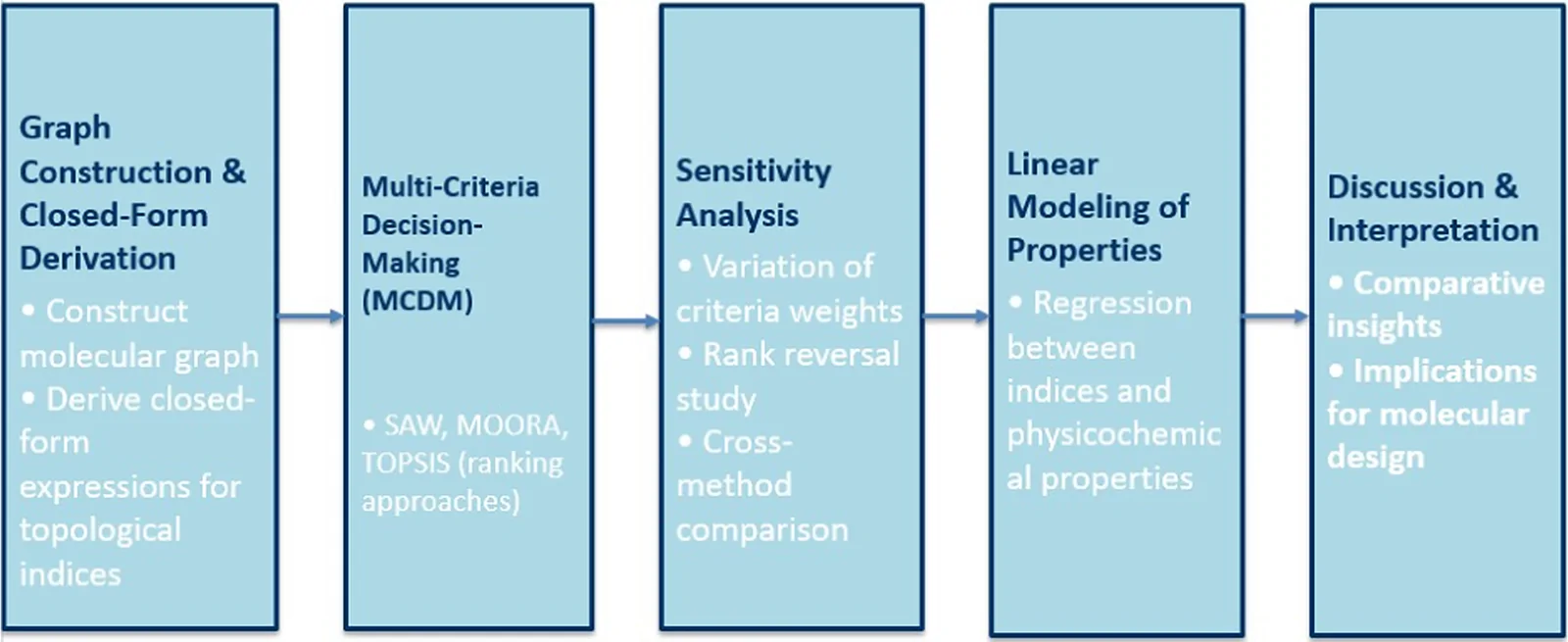

## Introduction

Dendrimers are unique nanostructures with a clearly defined, monodisperse, and uniform structure with branches that look like trees, low polydispersion, and high functionality. They have emerged as highly versatile nanocarriers for drug delivery and diagnostic applications and have attracted considerable research attention over the last few decades [1–3]. The physical and chemical properties of dendrimers are strongly governed by their molecular structure, which dictates key parameters such as solubility, encapsulation capacity, and surface reactivity [4, 5]. Owing to their distinctive structure–property relationships, dendrimers are considered ideal candidates for a wide range of applications in research, nanotechnology, and therapeutic delivery, including gene transfection, imaging, and drug transport [6]. Furthermore, advances in synthetic methodologies and an improved understanding of their biocompatibility and biodistribution have positioned dendrimers as promising platforms for clinical translation and pharmaceutical applications [7]. The development of large-scale and cost-effective commercial applications of dendrimer-based systems is expected to further enhance their technological and biomedical impact in the future [8].

Phosphorus-containing dendrimers have long been recognized as promising platforms for a wide range of biomedical applications, including drug delivery, gene transfection, and anticancer therapies [4, 6]. Scientists have developed phosphorus dendrimers for diverse biomedical applications, including gene delivery for acute lung injury, enzyme activation in Gaucher disease, and co-delivery of bioactive molecules for osteoarthritis treatment. They exhibit antibacterial activity against resistant Staphylococcus aureus, support protein delivery for anti-inflammatory therapy, and enhance tumor imaging due to their organized core-shell structure, underscoring their significant therapeutic potential [9–11]. Perylenediimide (PDI)-cored polyglycerol (*PG*) dendrimers have shown great promise in biomedical imaging and diagnostics. Earlier studies reported hybrid PDI-cored dendrimers with high quantum yields for live-cell imaging, water-soluble PDI dyes with nearly 100 percent fluorescence efficiency, and clickable dendrimers enabling target-specific protein labeling on bacterial and mammalian cells, thereby establishing important milestones in the development of fluorescent dendritic nanostructures [12].

Triazine dendrimers have demonstrated remarkable versatility in diverse biomedical applications. For example, a third-generation triazine dendrimer conjugated with polyethylene glycol (PEG) and paclitaxel exhibited significant in vitro and in vivo anticancer efficacy with reduced systemic toxicity compared to conventional formulations [13]. In another study, star-shaped triazine dendrimers were engineered to inhibit matrix metalloproteinase-9 (MMP-9) and vascular endothelial growth factor (VEGF), showing pronounced antiproliferative activity against cancer cells [14]. These findings collectively underline the therapeutic promise of triazine-based dendrimers as multifunctional platforms for targeted cancer treatment. The understanding of Aliphatic polyamide dendrimers with piperazine cores [15] described a synthetic approach for these dendrimers, reporting self-interruption at the fifth generation due to backfolding by peripheral groups. These dendrimers demonstrated resistance to enzymatic and hydrolytic degradation, along with antibacterial activity, indicating potential biological applications. Furthermore, [16] reviewed various synthesis strategies for poly-aliphatic amine dendrimers, including those with piperazine cores, and emphasized their promise in drug delivery and nanomedicine. The topological features of chemical structures have attracted increasing attention, particularly within the chemistry community. Numerous studies have incorporated the notion of topological structure into diverse chemical investigations. It is now well understood that the arrangement and connectivity of atoms within molecules exert a significant influence on many intrinsic physicochemical properties of molecular compounds. Systematic research on molecular descriptors, most of which are formulated through graph-theoretic principles, began in the early 1960 s, when various forms of such descriptors were proposed.

**Fig. 1:**
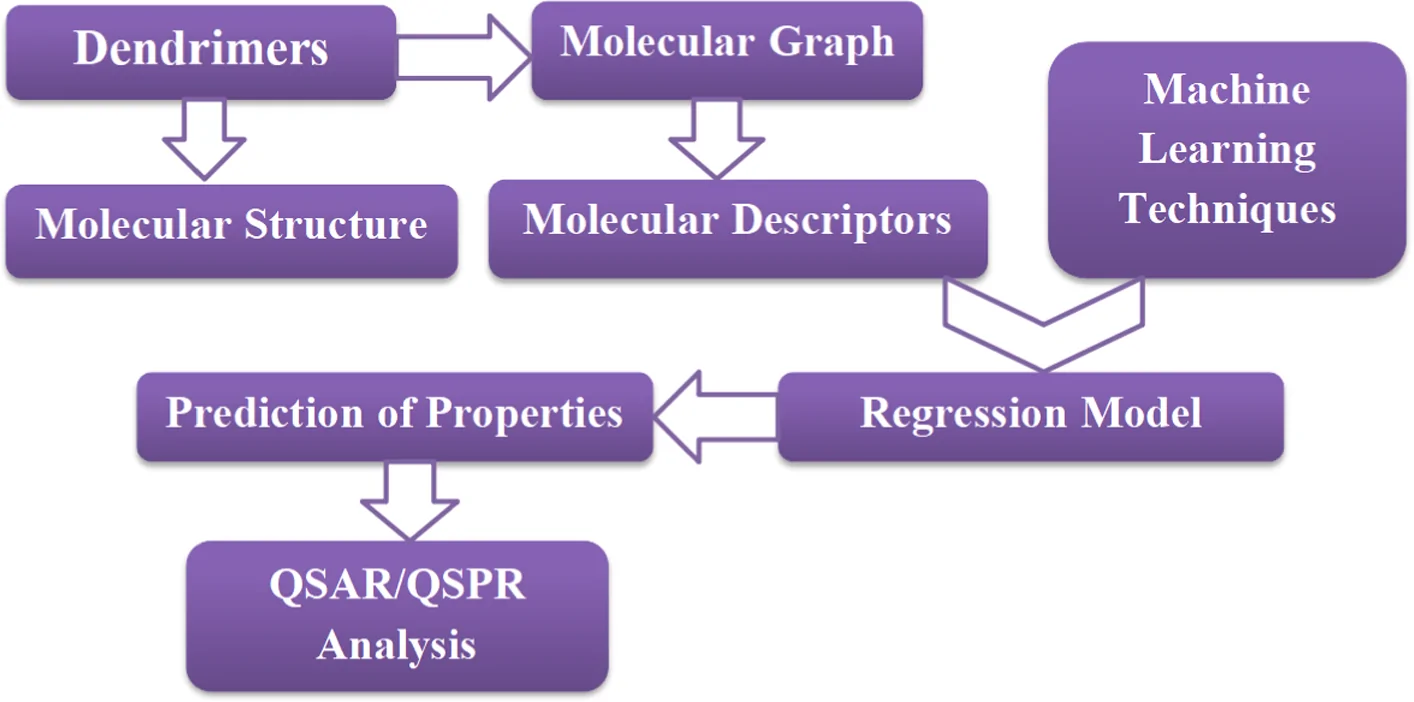
Schematic workflow showing the transformation of dendrimer molecular structures into topological descriptors and their subsequent use in machine learning and regression-based QSAR/QSPR models for predicting physicochemical and biological properties

By the late 1960 s, numerous structure–property relationships had been formulated, drawing not only on substituent effects but also on various indices designed to capture features of the complete molecular framework. These theoretical indices, often referred to as two-dimensional (2D) descriptors, were obtained from topological representations primarily through the use of graph-theoretic principles. Subsequent developments in Quantitative Structure-Activity Relationship (QSAR) and quantitative structure–property Relationship (QSPR) studies that relied on TIs were substantially influenced by the foundational contributions reported in [17, 18]. More recently, graph-theoretical methodologies have continued to evolve and have been integrated into modern computational applications. For example, M-polynomials [19] are employed to model biological control agents against Fusarium wilt, demonstrating the applicability of QSPR techniques that relate molecular structure to biological activity and improve predictive accuracy in plant disease management. The overall connection between molecular descriptors and QSAR/QSPR analyses is outlined in the flow chart shown in Fig. 1. In contemporary QSAR/QSPR studies, the derived molecular descriptors are employed as input features for statistical and machine learning techniques, including regression-based models, to establish quantitative relationships between molecular structure and physicochemical or biological properties. These computational approaches enable the prediction of molecular behavior from topological descriptors and facilitate the development of accurate predictive models for rational drug design, materials science, and nanomedicine. This relationship demonstrates the quantitative relationship between the physicochemical and biological properties of molecules and structural parameters like electronic, topological, and geometrical descriptors. Since it allows for the prediction of molecular behavior and the optimization of therapeutic efficacy, an understanding of these interactions is crucial for rational drug design and materials development.

Multi-criteria decision-making (MCDM) [20] refers to the process of selecting the best choice from a range of feasible alternatives based on several predetermined criteria. When making decisions in the real world, it is common to have to take into account multiple, sometimes contradictory, factors considered simultaneously. It enables systematic evaluation of factors/scenarios with a variety of frequently conflicting characteristics in scientific and technical applications [21]. Its applications include supply chain management, bridge risk assessment, material selection, and industrial environment optimization [22, 23]. These techniques help decision-makers compare several criteria that may differ in nature and measurement scale. The analytical framework and quality of input data have a major impact on the capacity to make logical and fair decisions in such situations. MCDM frameworks have recently been used in computational chemistry and drug development to find the best molecular candidates based on a variety of characteristics, including solubility, lipophilicity, molecular weight, and anticipated biological activity [20]. Bringing together MCDM with QSAR/QSPR analysis improves decision-making by offering a structured technique for evaluating various chemical alternatives based on their physicochemical and pharmacological features. Consequently, in modern computational and medicinal chemistry, MCDM is a potential decision-support tool for directing chemical prioritization, materials selection, and nanocarrier design. The primary goals of the current investigation are summarized as follows.

- Determine topological indices (TIs) based upon degree for the dendrimers under consideration.
- Examine the correlation between these indices and the physical and chemical features of derivatives of benzene.
- Rank dendrimers for drug delivery applications employing three MCDM techniques.
- Evaluate how stable and robust the rankings are to weight variations and rank reversal.
- Establish the mathematical framework to lead further experimental verification.

**Fig. 2:**
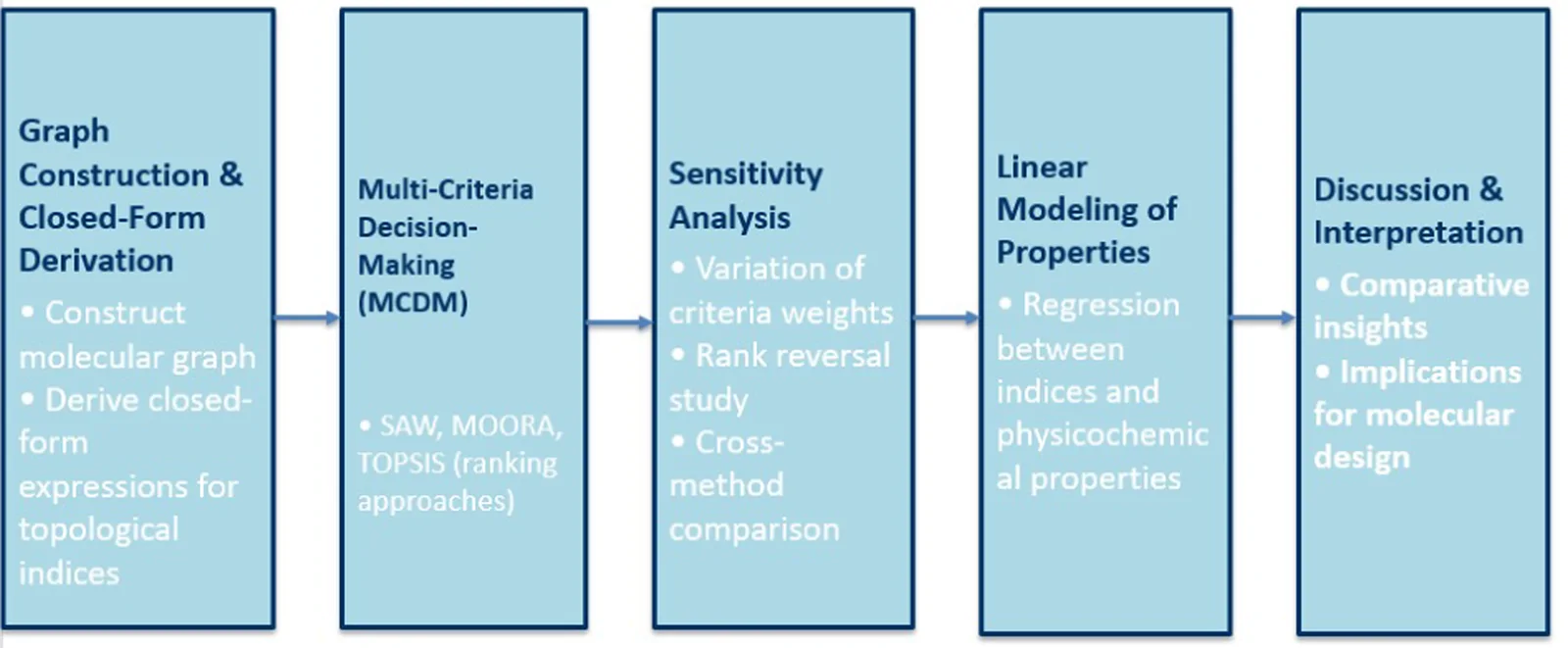
Workflow illustrating the main stages of the proposed study

The flowchart in Fig. 2 depicts the overall methodological framework of the study, including graph construction, MCDM analysis, sensitivity analysis, linear modeling of properties, and interpretation of the obtained results. In addition, sensitivity analysis is performed to provide reliable, robust, and interpretable decision outputs.

The remainder of this paper is organized as follows. Section 2 describes the materials and methods, Sect. 3 presents the computational results and closed-form expressions, Sect. 4 examines the correlation between TIs and physicochemical properties, Sect. 5 applies MCDM methods (SAW, TOPSIS, and MOORA), Sect. 6 focuses on stability and sensitivity analysis, Sect. 7 introduces linear modeling, Sect. 8 provides the discussion, and Sect. 9 concludes the study.

## Theoretical framework and methodology

Graph theory serves as a fundamental instrument across numerous academic disciplines, including coding theory, database systems, circuit design, secret sharing protocols, and various branches of theoretical chemistry. Cheminformatics, situated at the intersection of technology, graph theory, and chemistry, employs relevant graph invariants together with their corresponding molecular graphs to relate the structural features of organic molecules to their physicochemical behavior. A molecular graph consists of a collection of points and connecting lines that symbolize the atoms and covalent bonds of a molecule; in graph-theoretic terminology, these are referred to as vertices and edges, respectively [24]. The theoretical exploration of chemical structures through appropriate graph invariants has emerged as a compelling direction within mathematical chemistry, largely due to its usefulness in QSAR/QSPR investigations [25, 26]. TIs derived from such invariants are frequently utilized to estimate the physicochemical attributes of chemical compounds.

Throughout this paper, we denote a finite, simple, and connected molecular graph as *G*. *V*(*G*) and *E*(*G*) denote the vertex and edge set of *G*, respectively. In a molecular graph, the vertices correspond to atoms, while the edges represent the chemical bonds between atoms. The degree of a vertex v∈V(G), denoted by $d_{v}$, is defined as the number of edges incident to *v*. To establish a clear structural foundation for subsequent analysis, the dendrimer architectures considered in this study are modeled using graph theoretic representations. These graph models are constructed from the well-established chemical structures and generation-wise growth patterns of the corresponding dendrimers reported in the literature. Following the standard convention of chemical graph theory, hydrogen atoms are suppressed, and the remaining atoms and chemical bonds are represented by vertices and edges, respectively. Consequently, the inherent branching architecture and successive generation growth of the dendrimers are faithfully preserved in the resulting molecular graphs. To represent dendrimer structures, we define specific molecular graphs $D_i(n)$, where *n* indicates the generation stage and i=1,2,3,4 correspond to: $D_1(n)$ (Phosphorus-containing dendrimer), $D_2(n)$ (PDI-cored dendrimer), $D_3(n)$ (Triazine-based dendrimer), and $D_4(n)$ (Aliphatic polyamide dendrimer). These dendrimer-specific graph models form the structural basis for the computation and analysis of the degree-based topological indices. Since the degree of each vertex plays a fundamental role in characterizing molecular structure, degree-based topological indices are widely employed for quantifying structural properties of chemical compounds.

**Fig. 3:**
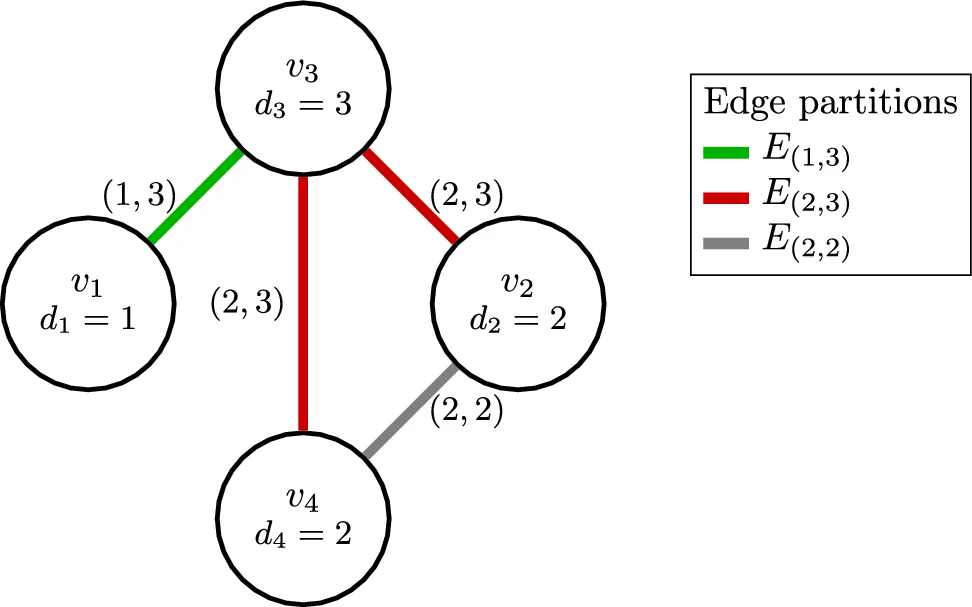
Schematic illustration of the degree-based partition of the edge set *E*(*G*) in a simple undirected molecular graph. Vertices are labeled by their degrees, and edges are grouped into partitions $E_{(d_u,d_v)}$ according to the degree pairs of their incident vertices

These indices depend not only on the degrees of individual vertices, but also on the degree pairs associated with edges. Figure 3 illustrates this concept through a simple schematic graph with four vertices labeled $v_1$ to $v_4$. Each vertex has a number below it indicating its degree (the number of edges incident to it). The edges are labeled and color-coded based on the degrees of the two vertices they connect. For example, an edge connecting a vertex of degree 1 and a vertex of degree 3 is labeled $E_{(1,3)}$ and colored green, while edges connecting degrees 2 and 3 are labeled $E_{(2,3)}$ and shown in red. The edge connecting two degree 2 vertices is labeled $E_{(2,2)}$ and colored gray. The legend on the right lists all the different degree pair types. These degree-based edge partitions $E_{(d_u,d_v)}$constitute the fundamental components used in the computation of degree-based topological indices, where summations are performed over all edges belonging to each partition.

Here, we focus on four widely studied degree-based indices: the F, AZI, NK, and NK$^*$ [27–33]. Their mathematical formulations are summarized in Table 1.

## Results

In this section, we present AZI, F, NK index, NK$^*$ index of phosphorus-containing dendrimers $D_1(n)$, PDI-cored dendrimers $D_2(n)$, Triazine-based dendrimers $D_3(n)$, and Aliphatic polyamide dendrimers $D_4(n)$.

**Table 1:** Structural descriptors of degree-based topological indices

Topological descriptors	Computational expressions
Augmented Zagreb index [27, 28]	$\text {AZI}(G) =\sum _{uv \in E(G)}\big ( \frac{d_{u}d_{v}}{d_{u}+d_{v}-2}\big ) ^3$
Forgotten index [30, 31]	$F(G) =\sum _{uv \in E(G)}(d_{u}^2+d_{v}^2)$
NK index [32, 33]	NK$(G) =\prod _{v \in V(G)}d_{v}$
NK$^*$ index [32, 33]	NK$^*(G) =\prod _{v \in V(G)}d_{v}^{d_{v}}$

**Fig. 4:**
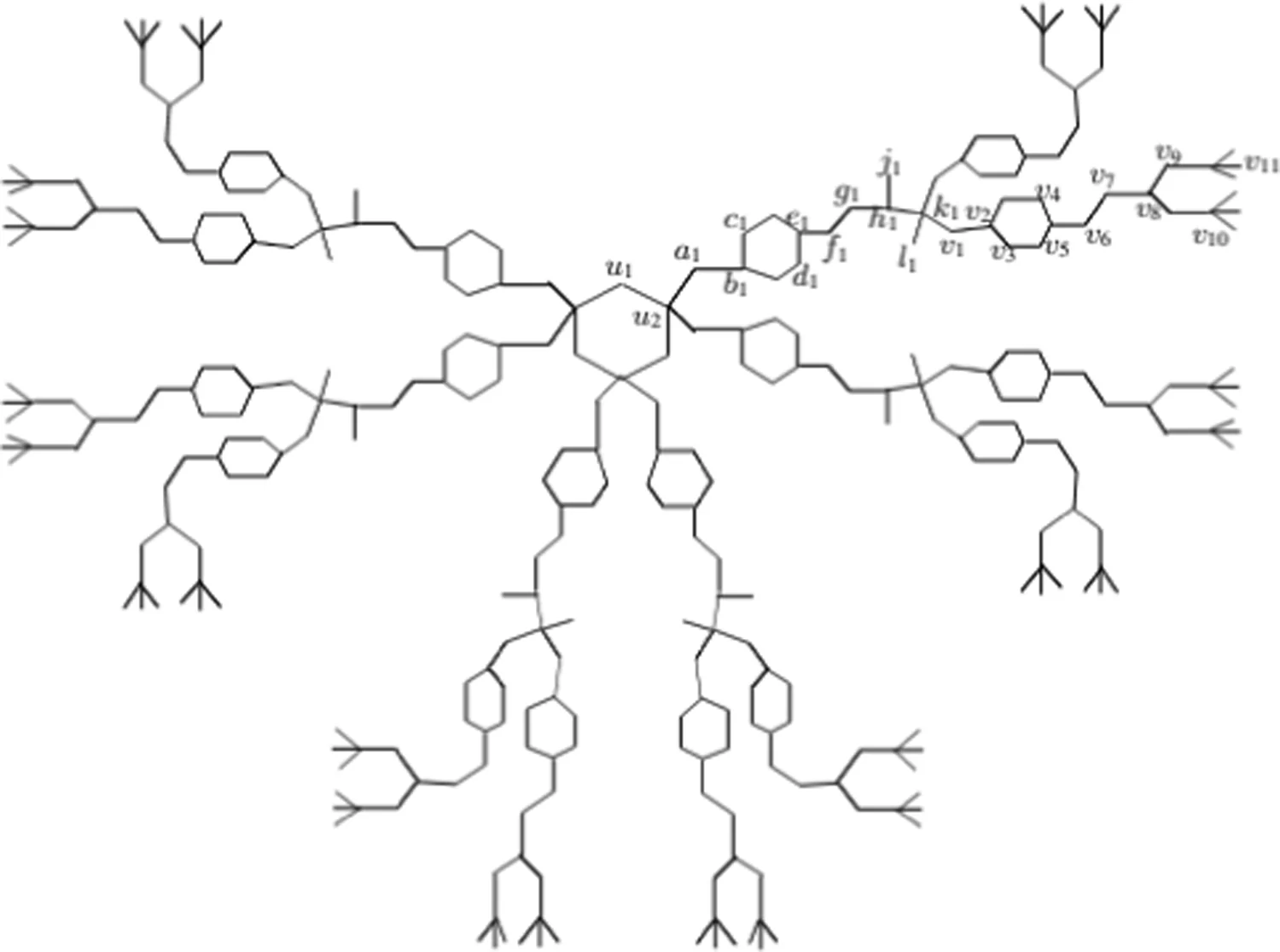
Schematic molecular graph $D_{1}(n)$ of the azabisphosphonate-functionalized phosphorus dendrimer (ABP dendrimer) at generation n=1, illustrating the degree-based edge partitions used in the computation of TIs. The frequencies of the corresponding edge classes are summarized in Table 2

### Phosphorus-containing dendrimers

Phosphorus-containing dendrimers possess functional groups with bonded nitroxyl radicals, and these radicals show a strong, favorable trading interaction [34]. In the present work, we consider the azabisphosphonate-functionalized phosphorus dendrimer (ABP dendrimer) [35]. The terminal azabisphosphonate groups are introduced through a post-synthetic surface functionalization step and are not present in the native phosphorus dendrimer architecture. This ABP-functionalized derivative was intentionally selected because of its excellent aqueous solubility and established relevance in drug delivery applications. The chemical structure of the ABP dendrimer is given in  [35]. The molecular graph $D_1(n)$, constructed from this chemical structure, is denoted by $D_1(n)$, where *n* represents the generation stage.

As the generation number *n* increases, the molecular graph $D_1(n)$ expands systematically through additional branching, representing higher dendrimer generations with increased numbers of vertices and edges. In Fig. 4, the vertices of the molecular graph of the ABP-functionalized phosphorus dendrimer $D_1(n)$ are labeled symbolically (e.g., $u_1, u_2, a_1, b_1, c_1, \ldots$) to distinguish vertices occupying different structural positions within the dendrimer architecture. An edge exists between two vertices whenever they are directly connected in the molecular graph. For instance, the edge connecting vertices $a_1$ and $b_1$ represents one occurrence of an edge whose end vertices have degrees $d_{a_1}=2$ and $d_{b_1}=3$. All edges connecting vertices with the same degree pair $(d_u,d_v)$ are grouped into the same edge class $E_{(d_u,d_v)}$ of $(D_1(n))$. The frequency of each edge class is obtained by counting the total number of such edges throughout the dendrimer graph. These frequencies are summarized in Table 2 and serve as the fundamental input for the computation of degree-based TIs. Thus, the vertex labeling in Fig. 4 visually illustrates how individual edges are identified, classified, and counted according to vertex degree pairs, while Table 2 provides the corresponding quantitative representation, and this procedure is carried out for all edge classes corresponding to representative vertex pairs in $D_1(n)$ to obtain the complete set of degree-based edge partitions. The order and size of $D_1(n)$ are $6(33 \times 2^{n} - 12)$ and $6(35 \times 2^{n} - 13)$, respectively [34], indicating that both the number of vertices (atoms) and edges (bonds) increase exponentially with *n*, consistent with the progressive branching of higher dendrimer generations.

*Result 1:* The closed-form expression for the Augmented Zagreb Index of $D_{1}(n)$ is given by

1 $$\begin{aligned} \text {AZI}(D_{1}(n)) = 2^{n}(1588.1931) - 597.4162. \end{aligned}$$

#### Proof

The AZI of a molecular graph G is defined as

2 $$\begin{aligned} \text {AZI}(G) = \sum _{uv \in E(G)} \left( \frac{d_u d_v}{d_u + d_v - 2}\right) ^3, \end{aligned}$$

where $d_u$ and $d_v$ denote the degrees of the adjacent vertices u and v, respectively. For the molecular graph $D_{1}(n)$, each distinct edge type $(d_u, d_v)$ and its corresponding frequency contribute separately to the total AZI value. Substituting each degree combination and its edge count into Equation (2) allows computation of the contribution from every edge category. For example, the contribution of edges of type (1,3) is given by

3 $$\begin{aligned} 6(2^{n}-1)\left( \frac{1\times 3}{1+3-2}\right) ^3= &   6(2^{n}-1)\left( \frac{3}{2}\right) ^3 \nonumber \\= &   6(2^{n}-1)\times 3.375 \nonumber \\= &   20.25(2^{n}-1). \end{aligned}$$

Similarly, for the edges of type (2,3),

4 $$\begin{aligned} 6(2^{n+4}-7)\left( \frac{2\times 3}{2+3-2}\right) ^3= &   6(2^{n+4}-7)\left( \frac{6}{3}\right) ^3 \nonumber \\= &   6(2^{n+4}-7)\times 8 \nonumber \\= &   48(2^{n+4}-7). \end{aligned}$$

The total AZI value is obtained by summing the contributions of all edge types

5 $$\begin{aligned} \text {AZI}(D_1(n)) = \sum _{(d_u,d_v)} [\text {edge count}] \times \left( \frac{d_u d_v}{d_u + d_v - 2}\right) ^3. \end{aligned}$$

After simplification, this expression reduces to the following closed-form equation

6 $$\begin{aligned} \text {AZI}(D_1(n)) = 2^{n}(1588.1931) - 597.4162. \end{aligned}$$

To maintain brevity, only the derivation for $D_{1}(n)$ is presented in full, while subsequent results follow the same computational procedure.

*Result 2:* Using the data from Table 2 in the Forgotten index formula, Equation (7) is evaluated for phosphorus-containing dendrimers

7 $$\begin{aligned} F(D_1(n)) = 2^{n}(3036) - 1002 \end{aligned}$$

*Result 3:* Based on the values in Table 3 and the NK index definition, Equation (8) is computed for phosphorus-containing dendrimers

8 $$\begin{aligned} \text {NK}(D_1(n)) = (2)^{12 \times 2^n} \times (3)^{4 \times 2^n + 16} \end{aligned}$$

**Table 2:** Edge partition of the molecular graph $D_{1}(n)$ for phosphorus-containing dendrimers

$(d_u,d_v)$	Edge count
(1, 3)	$$6(2^{n}-1)$$
(1, 4)	$$6(5\times 2^{n}-1)$$
(2, 2)	$$18(2^{n+1}-1)$$
(2, 3)	$$6(2^{n+4}-7)$$
(2, 4)	$$(3\times 2^{n+3})$$
(3, 4)	$$6(3\times 2^{n}-1)$$

Each $(d_{u}, d_{v})$ pair represents the degrees of connected vertices, and the edge count indicates their frequency

**Table 3:** Degree based vertex partition of the phosphorus-containing dendrimer

Representation	Degree	Frequency
$v_1$	1	$48 \times 2^{2n} - 12$
$v_2$	2	$96 \times 2^n - 39$
$v_3$	3	$36 \times 2^n - 18$
$v_4$	4	$18 \times 2^n - 3$

*Result 4:* Applying the values from Table 3 to the NK$^*$ index expression, Equation (9) is determined for phosphorus-containing dendrimers

9 $$\begin{aligned} \text {NK}^*(D_{1}(n)) = (2)^{96 \times 2^n - 78} \times (3)^{36 \times 2^n - 12} \times (4)^{18 \times 2^n - 3} \nonumber \\ \end{aligned}$$

**Fig. 5:**
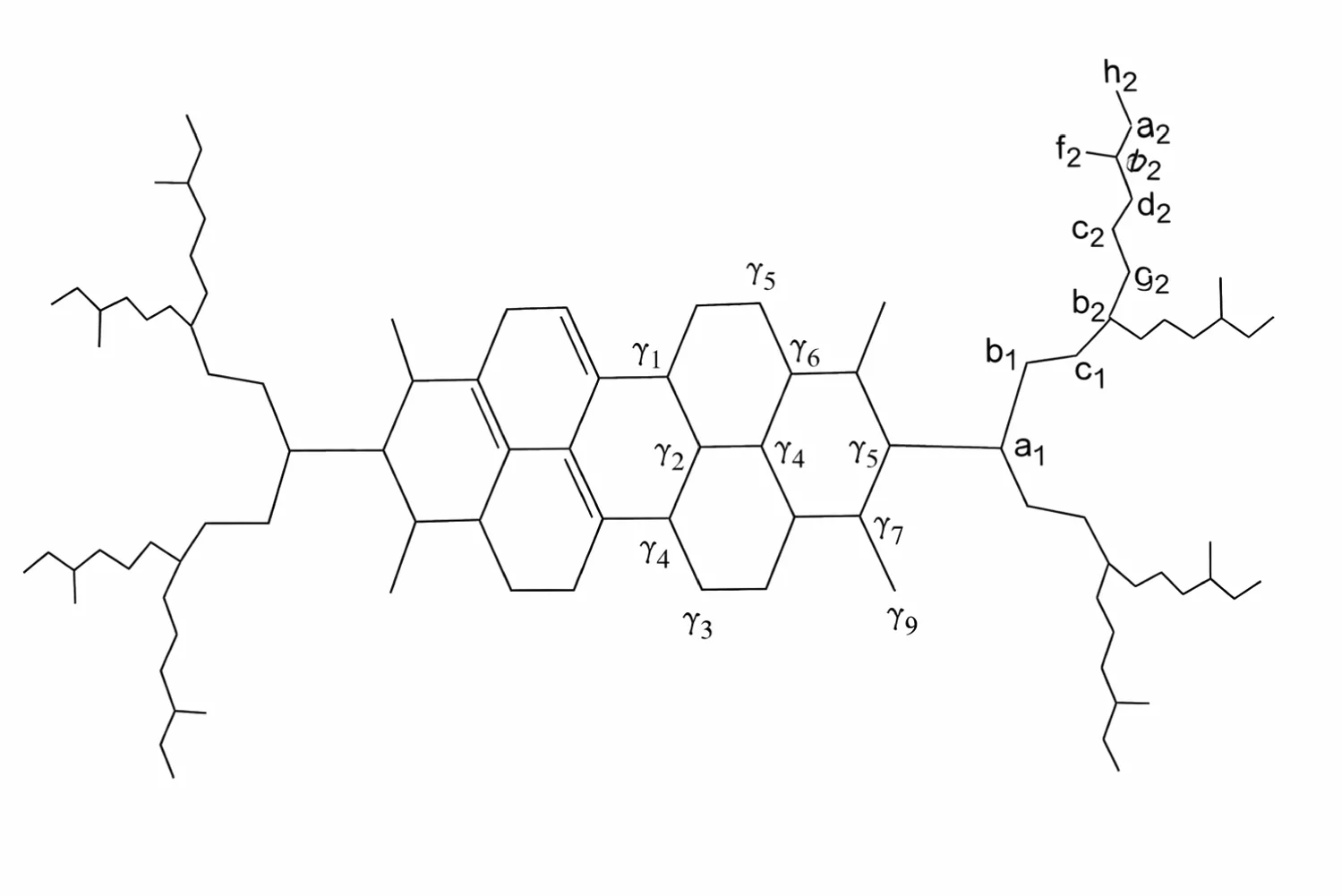
Schematic molecular graph $D_{2}(n)$ of a PDI-cored dendrimer at generation n=3, illustrating the atom-bond connectivity and branching pattern corresponding to the edge partition data in Table 4

### PDI-cored PG dendrimers

Water-soluble PDI-cored dendrimers possess several benefits, such as low cytotoxicity, intense red fluorescence, high quantum yield, good photostability, and easy surface modification. All these characteristics render them extremely suitable for numerous applications, such as fluorescence-based live-cell imaging and labeling [36, 37]. Let $D_2(n)$ denote the molecular graph of PDI-cored dendrimers, where *n* represents the generation stage. Figure 5 illustrates the molecular graph $D_2(n)$, which serves as the basis for our topological analysis. The corresponding edge partition of this graph is summarized in Table 4. In this representation, each vertex denotes an atom, and each edge represents a chemical bond; explicit atomic identities are not shown because the focus is on graph-theoretical connectivity rather than molecular geometry. As the generation number *n* increases, the molecular graph $D_2(n)$ expands through the addition of peripheral branching units, resulting in increased vertex count and edge density. This hierarchical growth reflects the progressive expansion of the dendrimer, where each new generation adds multiple terminal groups, enhancing its overall connectivity. Consequently, higher generations exhibit more complex topological structures with greater molecular size and branching diversity. Figure 5 thus provides the graph-theoretical connectivity model corresponding to the edge partition data in Table 4. The order and size of $D_2(n)$ are $20(2^n + 1)$ and 20×2n+26, respectively, indicating that both the number of vertices (atoms) and edges (bonds) grow systematically with the generation stage *n*, consistent with the dendrimer’s iterative molecular construction.

*Result 1:* Using the data from Table 4, the AZI for PDI-cored dendrimers $D_2(n)$ is determined as

10 $$\begin{aligned} \text {AZI}(D_2)(n) = (2^{n-2}) \times 611 + 272.09375 \end{aligned}$$

*Result 2:* Based on the values in Table 4 and the definition of the F-index, Equation (11) is evaluated for PDI-cored dendrimers as

11 $$\begin{aligned} F(D_2(n) = 218 \times 2^n + 444 \end{aligned}$$

*Result 3:* Employing the values from Table 5 along with the NK index formula, Equation (12) is derived for the PDI-cored PG dendrimer

12 $$\begin{aligned} \text {NK}(D_2(n))= &   2^{12 \times 2^n} \times 3^{4 \times 2^n + 16} \end{aligned}$$

**Table 4:** Degree-based edge partition of the molecular graph $D_{2}(n)$ for PDI-cored dendrimers

$(d_u,d_v)$	Edge Count
(1, 2)	$$2\times (2^{n+1})$$
(1, 3)	$$4\times (2^{n-1}+1)$$
(2, 2)	$$(3\times 2^{n+1}+1)$$
(2, 3)	$$(20\times 2^{n-1})$$
(3, 3)	(22)

Each $(d_{u}, d_{v})$ pair represents the degrees of connected vertices, and the edge count indicates their frequency

**Table 5:** Degree-based vertex partition of PDI-cored dendrimer

Representation	Degree	Frequencies
$v_1$	1	$4 \times 2^{2n}$+4
$v_2$	2	$12 \times 2^{n}$
$v_3$	3	$4 \times 2^{2n}$+16

*Result 4:* The NK$^*$ index for the PDI-cored PG dendrimer, as given in Equation (13), is computed by substituting the values from Table 5 into the corresponding expression:

13 $$\begin{aligned} \text {NK}^*(D_2(n)) = 2^{24 \times 2^n} \times 3^{12 \times 2^n + 48} \end{aligned}$$

### Triazine-based dendrimers

**Fig. 6:**
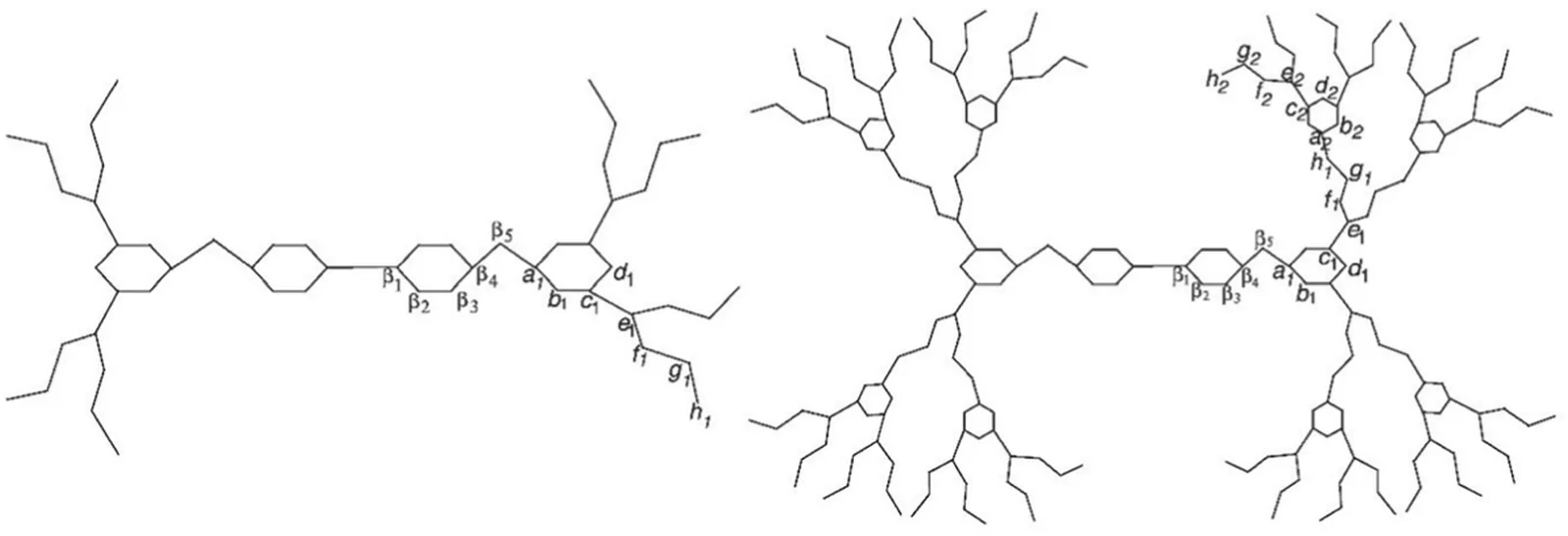
Schematic molecular graph $D_{3}(n)$ of a triazine-based dendrimer at generation n=2, illustrating the atom-bond connectivity and branching pattern corresponding to the edge partition data in Table 6

Triazine-based dendrimers are less toxic and can be further explored as drug carriers [13, 38]. Let $D_3(n)$ denote the molecular graph of a triazine-based dendrimer, where *n* represents the generation stage. Figure 6 illustrates the molecular graph $D_3(n)$, which serves as the foundation for the present topological analysis. In this representation, each vertex corresponds to an atom, and each edge represents a chemical bond. As the generation number *n* increases, the dendrimer expands through the successive addition of triazine branching units, leading to a systematic increase in both vertex and edge counts. This hierarchical growth reflects the iterative molecular construction characteristic of dendrimers, wherein each generation enhances overall branching and structural complexity.

The order and size of the molecular graph $D_3(n)$ are given by $\frac{2(5\times 2^{2n+2} + 1)}{3}$ and $(7\times 2^{2n+1} + 1)$, respectively. The corresponding edge partition of $D_3(n)$ is represented by $E(D_3(n))$, encompassing four distinct types of edges that define the topological diversity within the dendrimer structure. Table 6 provides a detailed classification of these edge subsets, while Fig. 6 offers the graph-theoretical connectivity model underlying this partition.

**Table 6:** Degree-based edge partition of the molecular graph $D_{3}(n)$ for triazine-based dendrimers, showing vertex degree pairs $(d_{u}, d_{v})$ and their edge counts

$(d_u,d_v)$	Edge count
(1, 2)	$$(2^{n+1})$$
(2, 2)	$$\frac{(5\times 2^{2n+1}-4)}{3}$$
(2, 3)	$$\frac{(11\times 2^{2n+1}-22)}{3}$$
(3, 3)	$$\frac{(2\times 2^{2n+1}-4)}{3}$$

*Result 1:* Using the data from Table 6 in the AZI formula, Equation (14) is evaluated for triazine-based dendrimers ($$ D_3(n)$$)

14 $$\begin{aligned} \text {AZI}(D_3(n)) = \left( \frac{5593}{48}\right) \times 2^{2n} - \frac{4057}{48} \end{aligned}$$

*Result 2:* Applying the values from Table 7 in the F-index expression, Equation (15) is computed for triazine-based dendrimers

15 $$\begin{aligned} F(D_3(n))= 156\times 2^{n} -130 \end{aligned}$$

*Result 3:* For triazine-based dendrimers, Equation (16) for the NK index is determined using the data in Table 7:

16 $$\begin{aligned} \text {NK}(D_3(n))= &   (2)^{8 \times 4^n} \times \left( 3 \right) ^{\frac{10 \times 4^n + 2}{3}} \end{aligned}$$

**Table 7:** Vertex partitioning of the triazine-based dendrimer based on vertex degree, illustrating the branching hierarchy and connectivity patterns of the molecule

Representation	Degree	Frequencies
$v_1$	1	$2^{2n+1}$
$v_2$	2	$8 \times 4^n$
$v_3$	3	$\frac{10 \times 4^n + 2}{3}$

*Result 4:* Using Table 7 and the NK$^*$ index formula, Equation (17) is calculated for triazine-based dendrimers

17 $$\begin{aligned} \text {NK}^*(D_3(n))= &   (2^{16 \times 4^n}) \times \left( 3^{10 \times 4^n + 2}\right) \end{aligned}$$

### Aliphatic polyamide dendrimers

**Fig. 7:**
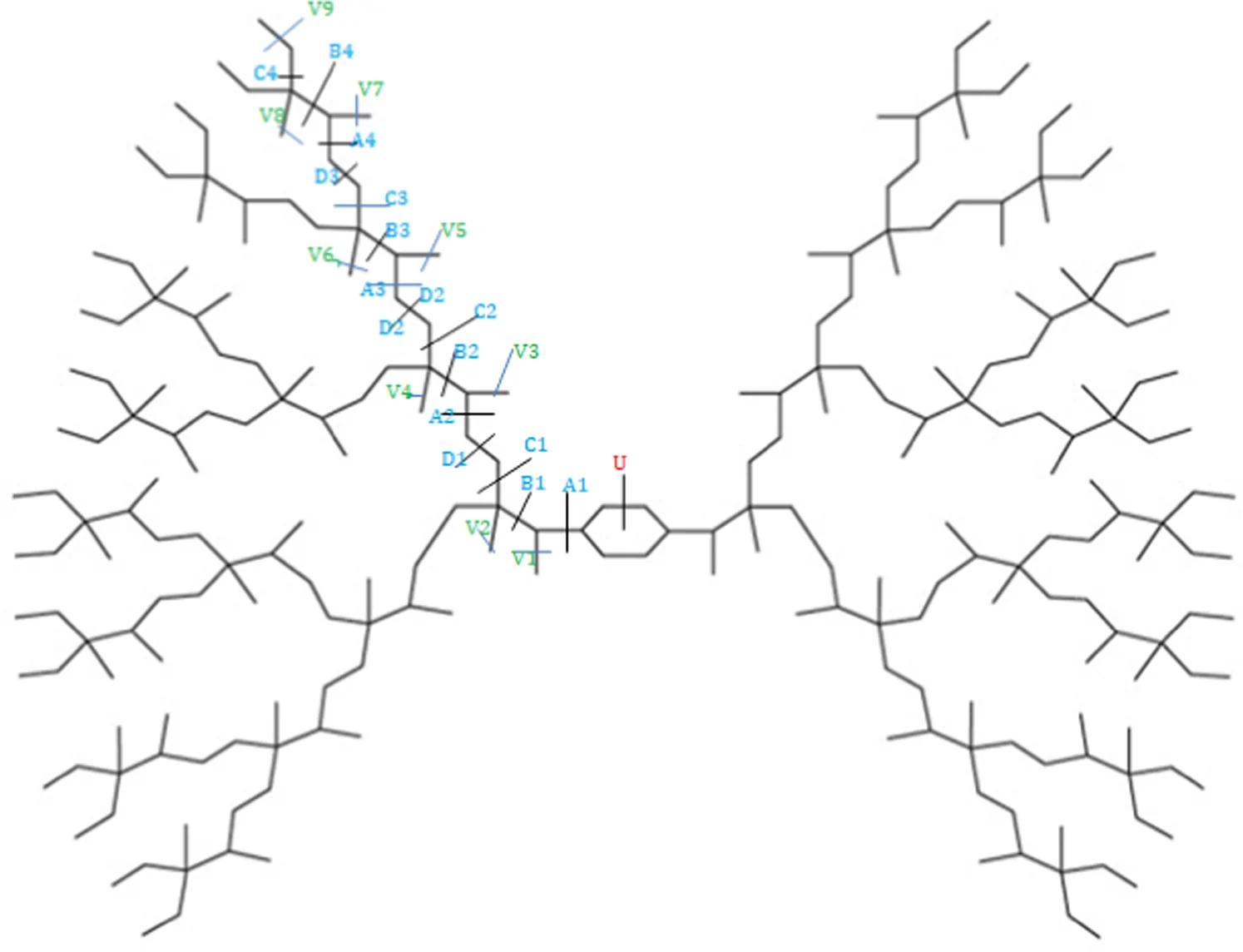
Schematic molecular graph of the Aliphatic polyamide dendrimer $D_4(n)$ at generation n=4, illustrating the branching hierarchy and connectivity patterns of the molecule

Jishkariani recently carried out the first research on aliphatic polyamide dendrimers with ethylenediamine and piperazine. These dendrimers are hydrolytically and enzymatically stable [15, 39]. Let $D_4(n)$ denote the molecular graph of the aliphatic polyamide-based dendrimer, where *n* represents the generation stage. Figure 7 illustrates the molecular graph $D_4(n)$, which forms the foundation for the present topological analysis. In this representation, each vertex corresponds to an atom, while each edge signifies a chemical bond. As the generation number *n* increases, the dendrimer expands through successive polyamide branching, resulting in a progressive increase in both vertex and edge counts. This iterative construction leads to a more complex and highly branched molecular architecture, reflecting the hierarchical nature of dendrimer growth. The order and size of the molecular graph $D_4(n)$ are given by $2(2^{n+3} - 5)$, respectively [39], indicating that the number of vertices and edges systematically increases with each generation. The corresponding edge partition of $D_4(n)$, denoted by $E(D_4(n))$, consists of four distinct types of edges that characterize the diverse bonding patterns within the dendrimer structure. A detailed classification of these edge subsets is presented in Table 8, while Fig. 7 provides the graph-theoretical connectivity model underlying this edge distribution.

**Table 8:** Degree-based edge partition of the molecular graph $D_{4}(n)$ for aliphatic polyamide dendrimers, showing vertex degree pairs $(d_{u}, d_{v})$ and their edge counts

$(d_u,d_v)$	Edge Count
(1, 2)	$$(2^{n+1})$$
(2, 3)	$$(2^{n+1})$$
(1, 3)	$$2(2^{n}-1)$$
(1, 4)	$$2(2^{n}-1)$$
(2, 2)	$$2(2^{n}-1)$$
(3, 4)	$$2(2^{n}-1)$$
(3, 3)	2
(2, 4)	$$(2^{n+2}-4)$$

*Result 1:* Using the data from Table 8 in the AZI formula, Equation (18) is evaluated for $D_4(m)$ dendrimers.

18 $$\begin{aligned} \text {AZI}(D_4(n))= &   \text {AZI}(D_4(n)) =2^{n} \times 119.1387414 \nonumber \\  &   + 64.35749 \end{aligned}$$

*Result 2:* Applying the values in Table 8 to the F-index expression, Equation (19) is computed for aliphatic polyamide dendrimers.

19 $$\begin{aligned} F(D_4(n)) =2^{n}\times 236-164 \end{aligned}$$

*Result 3:* For the aliphatic polyamide dendrimer, the NK index is calculated according to values in Table 9 and the definition of NK index, Eq. (20) can be computed as:

20 $$\begin{aligned} \text {NK}(D_{4}(n)) = (2)^{6\times 2^n-4} \times (3)^{2 \times 2^n}\times (4)^{2\times 2^n-2} \end{aligned}$$

**Table 9:** Degree-based vertex partition of the aliphatic polyamide dendrimer $D_4(n)$, illustrating the distribution of vertices according to their degrees and the branching structure of the molecule

Representation	Degree	Frequencies
$v_1$	1	$6\times 2^n-4$
$v_2$	2	$6\times 2^n-4$
$v_3$	3	$2\times 2^n$
$v_4$	4	$2\times 2^n-2$

*Result 4:* Using the values in Table 9 in the NK$^*$ index formula, Equation (21) is calculated for aliphatic polyamide dendrimers.

21 $$\begin{aligned} \text {NK}^*(D_{4}(n)) = 2^{12 \times 2^n-8} \times 3^{6\times 2^n }\times 4^{8 \times 2^n-8} \end{aligned}$$

The selected degree-based topological indices, AZI, F-index, NK index, and their revised versions are together indicative of critical structural features of dendrimers that determine their drug encapsulation and release properties. The value of the AZI indicates a general high level of molecular connectivity and compactness of the structure, and consequently, the larger the AZI, the higher the density of the molecular structure and the shorter diffusion routes, which is related to increased drug loading capacity and a decreased size effect of the release profile  [40]. The F-index is keen on high-degree vertices, which are associated with the branching points of the graph, which correlate with multivalency and surface functional groups, which are major determinants of drug conjugation efficiency and release modulation  [41]. The NK index, defined as the product of vertex degrees, reflects the trade-off between core and surface connectivity. This balance may enhance rigidity and stability, which are important factors in controlling drug release processes and improving resistance to early degradation. This is further narrowed down in the NK$^*$ index, which increases the degree of heterogeneity between core and periphery and highlights dendrimer architectures that support internal encapsulation rather than surface-based delivery. Although these indices give a precious topological understanding regarding structural features of interest to drug-delivery performance, they are strictly graph theoretical. They should, therefore, be combined with physicochemical and experimental information of dendrimer dynamics, their binding affinity, or their release kinetics to create a more elaborate understanding of dendrimer-mediated drug release [2, 42]. Therefore, in the following section, these descriptors are further investigated through their correlations with experimentally measurable physicochemical properties and subsequently employed in the MCDM framework.

## Relevance of topological indices to chemical properties

To investigate the relationships between topological descriptors and experimentally measurable physicochemical properties, a reference dataset of representative benzene derivatives was employed. Benzene-derived aromatic functionalities are frequently incorporated into dendrimer architectures and significantly influence their physicochemical behavior. The presence of such aromatic units can alter the physicochemical behavior of dendrimers, making them useful for applications like targeted drug delivery, molecular imaging, chemical sensing, and catalytic processes [43, 44]. Although the primary focus of this study is on dendrimers, comprehensive experimental physicochemical data are not uniformly available for all considered dendrimer families and generations. Therefore, representative benzene derivatives containing aromatic functional groups commonly encountered in dendrimer chemistry were employed as a reference dataset because their physicochemical properties are well documented in the literature. The correlations between these properties and the selected TIs were used to identify beneficial and non-beneficial descriptors and to guide the subsequent MCDM ranking of dendrimers. Thus, the benzene derivatives serve as a benchmark for descriptor evaluation rather than as alternative dendrimer systems.

**Table 10:** Computed topological indices for polycyclic aromatic hydrocarbons (benzene derivatives)

Molecule	AZI	F	NK	NK$^*$
Benzene	$4.80 \times 10^{1}$	$4.80 \times 10^{1}$	$6.40 \times 10^{1}$	$4.10 \times 10^{3}$
Naphthalene	$9.14 \times 10^{1}$	$1.18 \times 10^{2}$	$2.30 \times 10^{3}$	$4.78 \times 10^{7}$
Phenanthrene	$1.38 \times 10^{2}$	$1.88 \times 10^{2}$	$8.29 \times 10^{4}$	$5.57 \times 10^{11}$
Anthracene	$1.35 \times 10^{2}$	$1.88 \times 10^{2}$	$8.29 \times 10^{4}$	$5.57 \times 10^{11}$
Chrysene	$1.85 \times 10^{2}$	$2.58 \times 10^{2}$	$2.99 \times 10^{6}$	$6.50 \times 10^{15}$
Benzo[*a*]anthracene	$1.35 \times 10^{2}$	$1.88 \times 10^{2}$	$8.29 \times 10^{4}$	$5.57 \times 10^{11}$
Triphenylene	$2.32 \times 10^{2}$	$3.28 \times 10^{2}$	$1.07 \times 10^{8}$	$7.58 \times 10^{19}$
Tetracene	$1.78 \times 10^{2}$	$2.58 \times 10^{2}$	$2.99 \times 10^{6}$	$6.50 \times 10^{15}$

**Fig. 8:**
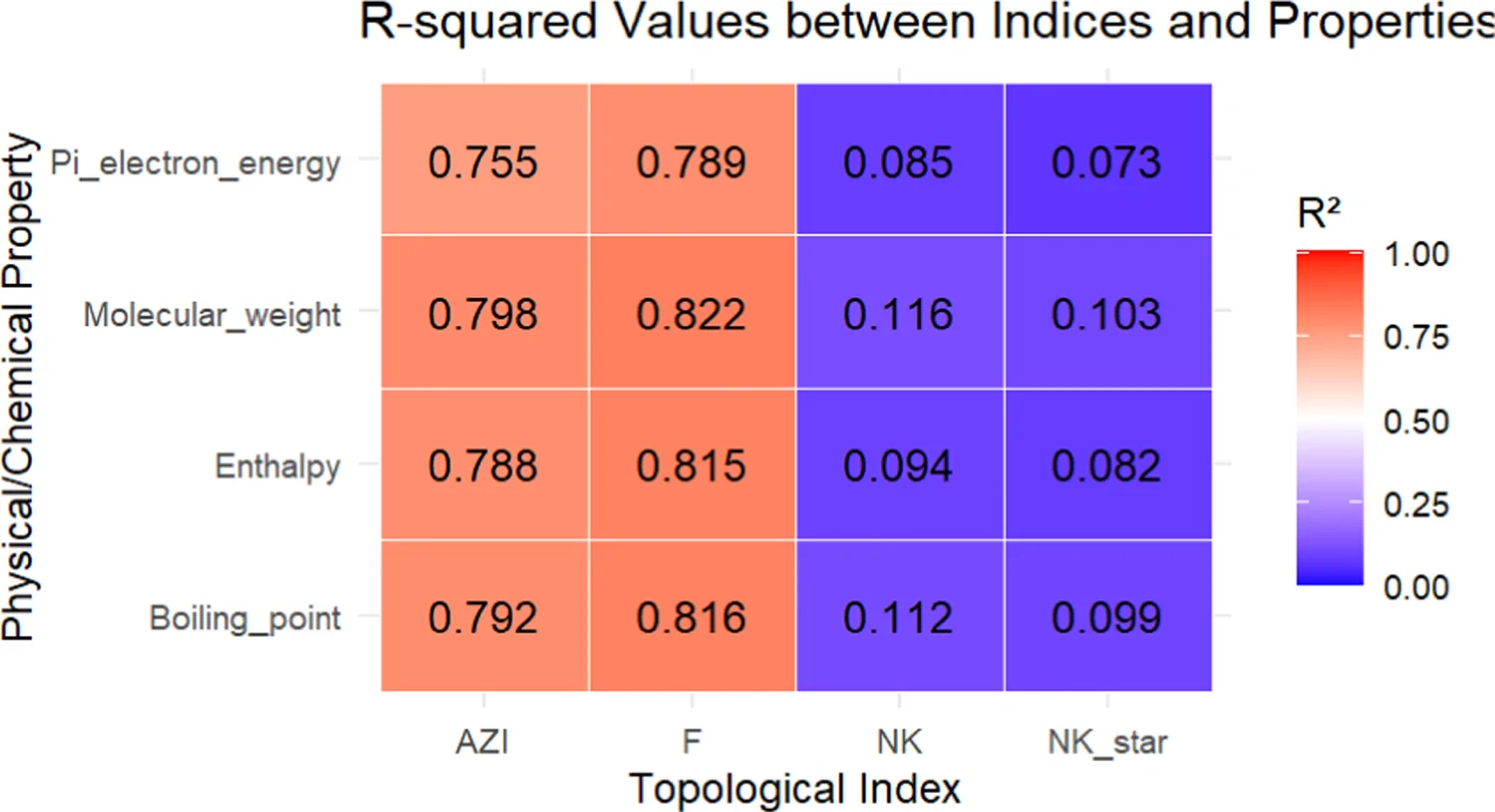
Heatmap of the correlation ($R^2$) values between the physicochemical properties of the reference benzene derivative dataset and the selected topological indices

Linear regression was performed to correlate the AZI, F index, NK, and NK$^*$ indices with the physicochemical properties of selected benzene derivatives. Eight aromatic compounds along with Benzene, Naphthalene, Phenanthrene, Anthracene, Chrysene, Benzo [a]anthracene, Triphenylene and Tetracene and their topological indices are given in Table 10. Table 11 contains the experimental values of boiling point, enthalpy, total π-electron energy, and the molecular weight, and Fig. 8 summarizes the values of $R^2$ of each correlation, or more precisely, the coefficient of determination. The regression indicates that the predictive performance of different indices shows a significant variation, with the strongest and the most stable correlation being exhibited by AZI and F-Index. The F-index was found to be best correlated, and an $R^2$ of molecular weight is 0.822, and this shows that it is able to explain more than 82 percent variation in the data. The AZI index has also been doing well with a maximum of $R^2$ of 0.798 with the same property and 0.788 with enthalpy. These high correlations show that the molecular properties strongly depend on the local vertex degrees (atom connectivity) in the molecular graph which these indices effectively reflect.

In contrast, the NK and its NK$^*$ proved to be ineffective descriptors for this chemical family, with $R^2$ values consistently negligible, ranging from 0.073 to 0.116. This poor performance aligns with observations for polycyclic aromatic hydrocarbons (PAHs), which are planar molecules composed of fused benzene rings characterized by uniform carbon connectivity and limited structural variation [45]. Such uniformity limits the sensitivity of multiplicative vertex degree functions, resulting in weak correlations with molecular properties. In contrast, dendrimers, particularly phosphorus-containing and PDI-based types, exhibit heterogeneous branching, variable coordination environments, and diverse electronic structures. These features, including heteroatom incorporation and conjugated linkages, generate complex connectivity patterns and enhance the responsiveness of degree-based indices. Consequently, dendrimers provide a more suitable and diverse model than PAHs, allowing indices like the AZI and F-index to better capture structural variations and predict molecular properties in electronically and topologically rich systems.

Conversely, degree-based indices such as the AZI and the F-index exhibit stronger correlations because they are directly derived from local bonding environments and overall network connectivity. The AZI considers the ratio of vertex degrees between bonded atoms, thereby emphasizing local bonding asymmetry, while the F-index incorporates the squared degree of each vertex, effectively capturing global connectivity within the molecular structure. These mathematical formulations make both indices more sensitive to variations in bond distribution and molecular branching compared to NK-type models, which rely on simple multiplicative degree functions and therefore overlook subtle differences in atomic coordination.

The $R^2$ analysis in Fig. 8 therefore provides empirical support affirming the AZI and F-index as reliable tools for predicting molecular properties across complex aromatic and dendritic systems.

**Table 11:** Physicochemical properties of representative benzene derivatives retrieved from PubChem [46] and used as a reference dataset in the descriptor analysis

Structure	Boiling point	Enthalpy	π-electron energy	Molecular weight
Benzene	78.1	80.1	75.2	8
Naphthalene	128.17	218	140.0	13.683
Phenanthrene	178.23	338	202.7	19.448
Anthracene	178.23	340	222.6	19.314
Chrysene	228.3	431	271.1	25.192
Benzo[a]anthracene	228.3	425	277.1	25.101
Triphenylene	228.3	429	275.1	25.275
Tetracene	228.3	440	310.5	25.188

## Multiple criteria decision-making technique

Multi-objective decision-making (MODM) and multi-attribute decision-making (MADM) [47] represent the two principal categories within the broader framework of multiple-criteria decision-making (MCDM) [48]. Over the past decade, MCDM has emerged as a rapidly expanding research area, providing robust methodologies to address complex decision problems across various fields, including industrial engineering. Each MCDM technique possesses distinct advantages and employs different computational frameworks to handle benefit and cost criteria, as well as diverse normalization approaches.

The literature reports a variety of well-established methods, each suited to particular types of problems. These include the Analytic Hierarchy Process (AHP) [49] for structuring complex decisions, SAW [50] for its straightforward weighted-sum approach, and distance-based techniques like the TOPSIS [47] and Gray Relational Analysis (GRA). Other prominent methods include Data Envelopment Analysis (DEA) for efficiency measurement, the PROMETHEE method [51] for outranking, and MOORA [52] for optimizing competing objectives. Fuzziniess has also been comprehensively incorporated to extend the applicability [53, 54].

**Table 12:** Classification of criteria into beneficial and non-beneficial types, indicating which factors positively or negatively influence the dendrimer rankings

Index	Property	Beneficial/Non-Beneficial
AZI index	Enthalpy	Beneficial
Forgotten index	Boiling point	Beneficial
NK index	π-electron energy	Non-Beneficial
NK$^*$ index	Molecular weight	Non-Beneficial

**Table 13:** Decision matrix showing the evaluation of the first-generation (n=1) dendrimer alternatives across all considered criteria, used as input for the MCDM analysis

Dendrimers	AZI index	F index	NK	NK$^*$
Phosphorus-containing	2578.97	5070	$4.7384 \times 10^{18}$	$6.4964 \times 10^{82}$
PDI-cored	577.5938	880	$4.7384 \times 10^{18}$	$6.3412 \times 10^{48}$
Aliphatic polyamide	302.6350	308	$3.3178 \times 10^{5}$	$2.2833 \times 10^{15}$
Triazine-based	381.5625	182	$2.0543 \times 10^{16}$	$2.0184 \times 10^{39}$

**Table 14:** Normalized decision matrix of dendrimer alternatives, showing scaled values of all criteria for use in the MCDM ranking analysis

Dendrimers	AZI index	F index	NK index	NK$^*$ index
Phosphorus-containing	0.671474	0.787267	0.498918	1.0
PDI-cored	0.150385	0.136646	0.498918	0
Aliphatic polyamide	0.078796	0.047826	0	0
Triazine-based	0.099346	0.028261	0.002163	0

In order to use such MCDM methods, we separate beneficial and non-beneficial criteria using four major physicochemical features of dendrimer structures, namely, molecular weight, enthalpy, boiling point, and π-electron energy. These parameters were correlated with their corresponding topological indices in terms of numerical values. The entropy approach was used to derive objective weight values of each of the properties based on the situation where overall weight is an absolute one to ensure a rigorous and data-driven basis for the analysis of the observed decisions, which immediately follows. The assignment of each property to a specific topological index in Table 12 was guided by their highest observed correlation coefficients (or more precisely coefficients of determination $R^2$ values) as illustrated in Fig. 6. This selective matching brings out the most representative and chemically significant relationships between molecular properties and structural indices, and eliminates redundancy in the less significant correlations, which gives a clear and data-supported foundation to the subsequent decision analysis.

### Entropy method

The entropy weighting technique is a well-established approach in MCDM and has been extensively applied for objective weighting in chemical graph theory and QSPR / QSAR modeling [55]. It measures the degree of variability or dispersion within the data, where higher variability indicates that a particular criterion provides more discriminative information and, therefore, should receive a greater weight. Conversely, if the values of a criterion show little variation, its contribution to the overall decision process is considered lower, and its assigned weight is reduced.

The computation involves the following steps:

1. *Decision matrix* The decision matrix for the four dendrimer families (Phosphorus-containing, PDI-cored, Aliphatic polyamide, and Triazine-based) for n=1 generation was constructed using the selected topological indices: AZI, F-index, NK, and NK$^*$. The decision matrix is given below in Table 13.
2. *Normalization* The decision matrix is first normalized to ensure fair comparison across criteria with different units or scales, preventing large values like NK and NK$^*$ from dominating the rankings. The normalized value $q_{kl}$ for the *k*th alternative under the *l*th criterion is calculated as
  22 $$\begin{aligned} q_{kl} = \frac{x_{kl}}{\sum _{k=1}^{r} x_{kl}}, \end{aligned}$$
  where $x_{kl}$ is the original value of the decision matrix for the considered index. Table 14 presents the normalized decision matrix, allowing each index to contribute proportionally and reflect structural significance rather than absolute magnitude.
3. *Entropy calculation* For each criterion, the entropy value $E_{l}$ is computed using:
  23 $$\begin{aligned} E_{l} = -m \sum _{k=1}^{r} q_{kl} \ln (q_{kl}), \end{aligned}$$
  where $m = \frac{1}{\ln (r)}$ ensures normalization and *r* denotes the number of alternatives. Using the above equation, we compute the entropy measure of project outcomes in Table 15.
4. *Determining criterion weights* The weight $w_{l}$ for each criterion is obtained as:
  24 $$\begin{aligned} w_{l} = \frac{1 - E_{l}}{\sum _{l=1}^{s} (1 - E_{l})}, \end{aligned}$$
  where *s* is the total number of criteria. This ensures that the sum of all weights equals one. Based on the entropy notion, we define the objective weight in Table 16.

**Table 15:** Entropy values of the topological indices, used to determine the relative weights of criteria in the MCDM analysis

Topological index	Entropy
AZI	0.708337
Forgotten Index	0.509607
NK	0.510052
NK$^*$	≈ 0.0000

**Table 16:** Target weights of the criteria, derived from entropy analysis, used to prioritize the importance of each topological index in the MCDM ranking

Dendrimer	AZI	Forgotten	NK	NK$^*$
Phosphorus-containing	0.6715	0.7873	0.4989	1.0000
PDI-cored	0.1504	0.1366	0.4989	$9.76 \times 10^{-35}$
Aliphatic polyamide	0.0788	0.0478	$3.49 \times 10^{-14}$	$3.51 \times 10^{-68}$
Triazine-based	0.0993	0.0283	$2.16 \times 10^{-3}$	$3.11 \times 10^{-44}$
$W_l$	0.128372	0.215841	0.215646	0.440140

### SAW method

**Fig. 9:**
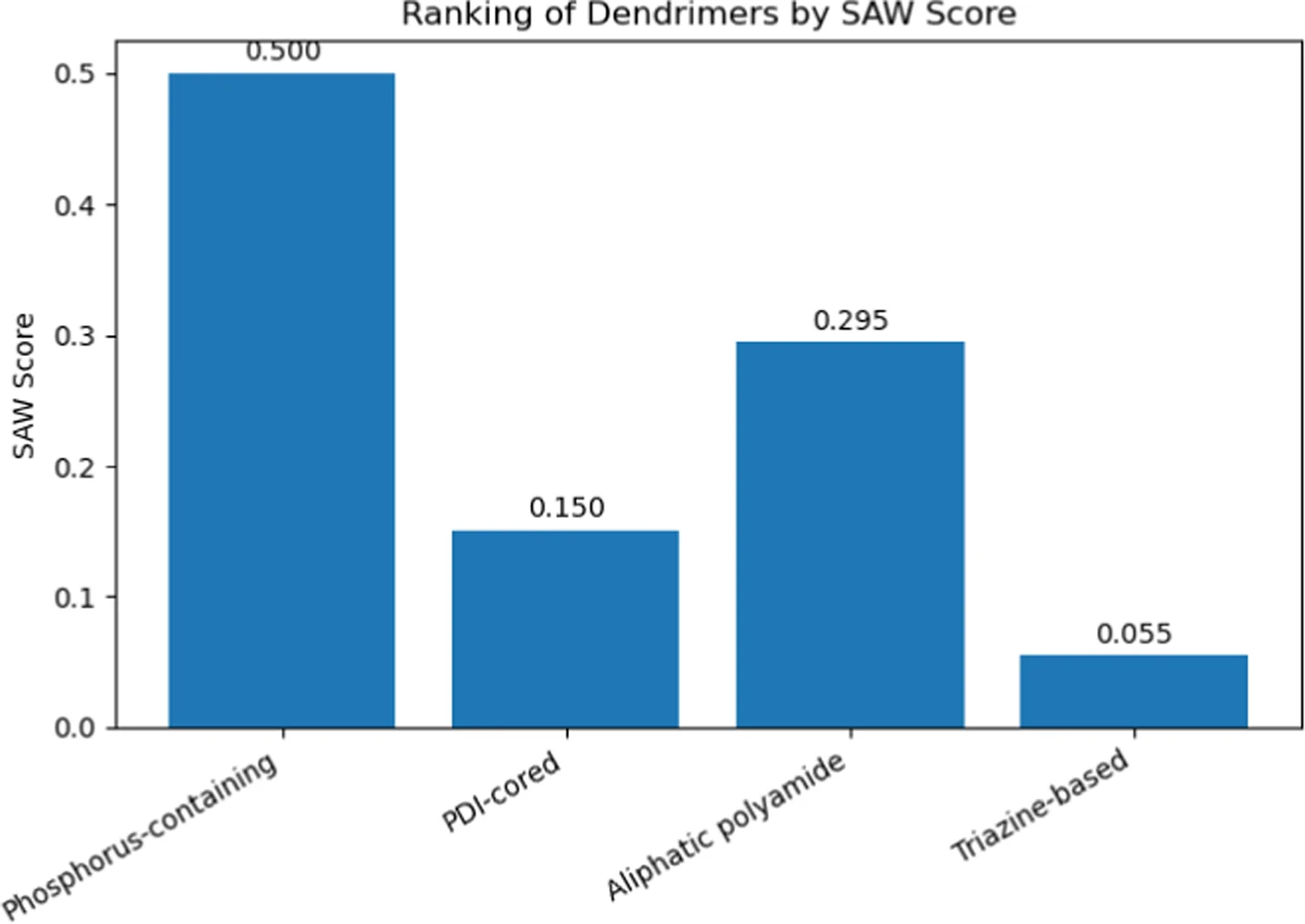
SAW scores and resulting rankings of dendrimer alternatives based on the weighted evaluation criteria

The SAW technique is a typical and convenient method of MCDM  [47, 50]. In this approach, each option is assigned a score computed as the sum of the weighted values of all criteria. Criteria that exhibit greater variation across the alternatives provide more discriminative information and are therefore assigned higher importance. Conversely, criteria with little variation receive lower weights and have less influence on the final decision. This is why the SAW approach is a simple method of integrating various topological indices and comparing molecular graphs with one another effectively. The computation involves the following steps:

1. *Normalization of the decision matrix* The initial step of the SAW technique is transforming the original decision matrix into a dimensionless form. This is done through normalization, which ensures that the criteria measured in different units can be directly compared. The most popular normalization method is the Max method, which is used because of its simplicity and efficiency. This technique is most appropriate in our analysis since it scales various criteria to a comparable scale, hence making it easy to make a multi-criteria decision. During normalization, it is important to differentiate between benefit (advantageous) and cost (non-beneficial) criteria: For benefit criteria, where higher values indicate better performance, each element is divided by the maximum value in its column. For cost criteria, where lower values are preferred, the minimum value in the column is divided by each element. The normalization formula is expressed as:
  25 $$\begin{aligned} x_{kl} = {\left\{ \begin{array}{ll} \dfrac{x_{kl}}{\max (x_l)}, &  \text {if } l \in A_{\text {benefit}} \\ \dfrac{\min (x_l)}{x_{kl}}, &  \text {if } l \in A_{\text {cost}}, \end{array}\right. } \end{aligned}$$
  where $x_{kl}$ is the normalized value for the *k*-th alternative with respect to the *l*-th criterion, $\max (x_l)$ and $\min (x_l)$ are the maximum and minimum values of criterion *l*, respectively, $A_{\text {benefit}}$ and $A_{\text {cost}}$ are the sets of benefit and cost criteria. The original decision matrix is shown in Table 13, and the normalized decision matrix is presented in Table 17. Assign weight *V* to each criterion $V = [v_1,v_2,v_3,....,v_r].$ In Table  18, we have allocated the weights to chemical/physical properties
2. *Ranking scores* After the normalization and weight assignment, the final ranking score for each alternative is computed by summing the weighted normalized values across all criteria. The formula used in the SAW method is:
  26 $$\begin{aligned} A_k = \sum _{l=1}^{r} v_l \cdot x_{kl}, \end{aligned}$$
  where $A_k$ is the final score of the *k*-th alternative, $v_l$ is the weight of the *l*-th criterion, $x_{kl}$ is the normalized performance value of the *k*-th alternative with respect to the *l*-th criterion, and *r* is the total number of criteria. The alternatives are then ranked in descending order of $A_k$, with the highest score indicating the most preferred option. The SAW ranking scores for the evaluated dendrimers are presented in Table 19 and illustrated in Fig. 9, showing the relative performance of each alternative based on the weighted normalized criteria.

**Table 17:** Normalized decision matrix used in the SAW method for dendrimer ranking

Dendrimers	AZI index	F index	NK index	NK$^*$ index
Phosphorus-containing	1.0000	1.0000	$7.0042 \times 10^{-14}$	$3.5152 \times 10^{-68}$
PDI-cored	0.2239	0.1736	$7.0042 \times 10^{-14}$	$3.1832 \times 10^{-35}$
Aliphatic polyamide	0.1174	0.0608	1.0000	1.0000
Triazine-based	0.1480	0.0359	$1.6140 \times 10^{-11}$	$8.9992 \times 10^{-44}$

**Table 18:** Weights of topological indices used in the SAW method for dendrimer ranking

Dendrimers	AZI index	F index	NK index	NK$^*$ index
Phosphorus-containing	1.0000	1.0000	$7.0042 \times 10^{-14}$	$3.5152 \times 10^{-68}$
PDI-cored	0.2239	0.1736	$7.0042 \times 10^{-14}$	$3.1832 \times 10^{-35}$
Aliphatic polyamide	0.1174	0.0608	1.0000	1.0000
Triazine-based	0.1480	0.0359	$1.6140 \times 10^{-11}$	$8.9992 \times 10^{-44}$
Weightage	0.25	0.25	0.25	0.25

### MOORA method

The MOORA (Multi-Objective Optimization by Ratio Analysis) method is a widely used MCDM approach for ranking alternatives based on multiple criteria [47, 52]. It evaluates each alternative by comparing normalized values of beneficial (the higher the better) and non-beneficial (the lower the better) criteria. The normalized values are combined to calculate an overall performance score for each alternative. Alternatives with higher scores are considered better. MOORA is suitable for dendrimer evaluation due to its ability to handle multiple conflicting criteria with different scales and physical meanings in a transparent normalization framework. In dendrimer selection, structural descriptors, topological indices, and physicochemical properties often exhibit competing effects on drug delivery performance. MOORA efficiently balances such criteria by simultaneously maximizing beneficial attributes (e.g., branching efficiency and structural stability) while minimizing non-beneficial ones, thereby providing a robust and interpretable ranking. The MOORA method includes the following steps:

**Table 19:** SAW scores and corresponding rankings of dendrimer alternatives based on the weighted evaluation criteria

Rank	Alternative	SAW score
1	Phosphorus-containing	0.500000
2	PDI-cored	0.150375
3	Aliphatic polyamide	0.294550
4	Triazine-based	0.054975

**Table 20:** Column-wise Euclidean normalized decision matrix used in the MOORA method, showing scaled values of dendrimer alternatives across all evaluation criteria

Dendrimers	AZI index	F index	NK index	NK$^*$ index
Phosphorus-containing	0.9597	0.9829	0.7071	1.0000
PDI-cored	0.2149	0.1706	0.7071	$9.7611 \times 10^{-35}$
Aliphatic polyamide	0.1126	0.0597	$4.9511 \times 10^{-14}$	$3.5147 \times 10^{-68}$
Triazine-based	0.1420	0.0353	$3.0656 \times 10^{-3}$	$3.1070 \times 10^{-44}$

**Fig. 10:**
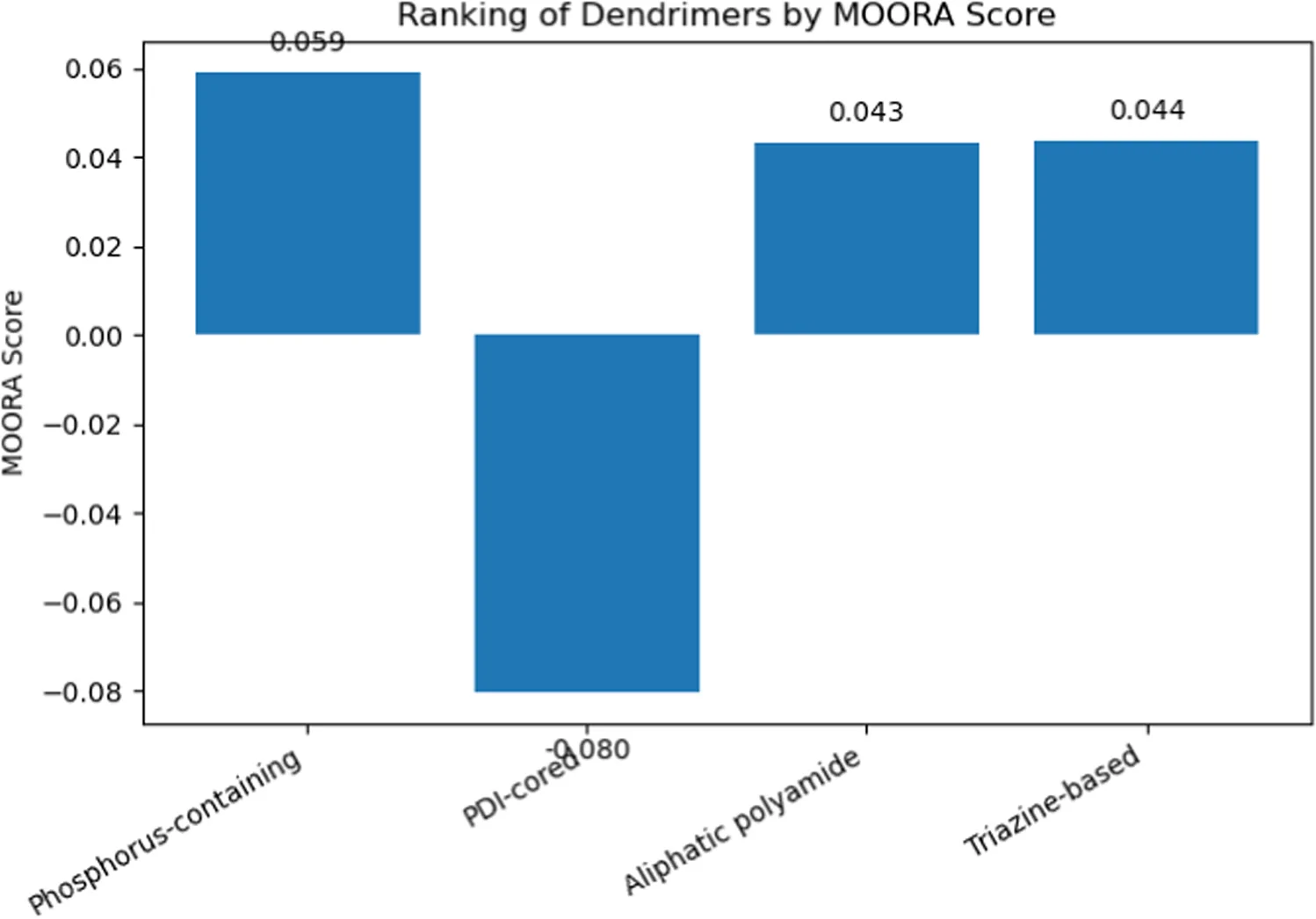
MOORA scores and corresponding rankings of dendrimer alternatives based on the weighted evaluation criteria

1. * Create decision matrix* At first, we create a decision matrix shown in Table 13.
2. * Normalize the decision matrix* This step converts the initial decision matrix into a dimensionless form, allowing all criteria to be compared on a common scale.
  27 $$\begin{aligned} x^{*}_{kl} = \frac{x_{kl}}{\sqrt{\sum _{k=1}^{q} x_{kl}^2}}, \qquad l = 1, 2, \ldots , r, \end{aligned}$$
  where $x_{kl}$ is the normalized value for the *k*-th alternative with respect to the *l*-th criterion. Using Eq. (27), we normalize the decision matrix as shown in Table 20.
3. * Attribute optimization* After obtaining the normalized decision matrix, the next step is to combine the attribute values according to whether they represent beneficial (maximization) or non-beneficial (minimization) criteria, as in Table 12. For a problem with *g* beneficial attributes and (r-g) non-beneficial attributes as, the overall performance score for the *k*-th alternative is computed as:
  28 $$\begin{aligned} a_k = \sum _{l=1}^{g} x^{*}_{kl} - \sum _{l=g+1}^{r} x^{*}_{kl} \end{aligned}$$
  where *g* is the number of beneficial criteria, (r-g) is the number of non-beneficial criteria and $a_k$ is the aggregated normalized score for alternative *k*. In many decision-making cases, certain attributes carry more importance than others. To account for this, each attribute is multiplied by its assigned weight $v_l$, leading to the weighted expression:
  29 $$\begin{aligned} a_k = \sum _{l=1}^{g} v_l\,x^{*}_{kl} - \sum _{l=g+1}^{r} v_l\,x^{*}_{kl}, \quad l = 1, 2, \dots , r. \end{aligned}$$
  Depending on the specific mix of beneficial and non-beneficial criteria, as in Table 12, the value of $a_k$ can be positive or negative. Alternatives are ranked in descending order of $a_k$, with the highest score indicating the most preferred option and the lowest score the least preferred, as shown in Table 21 and illustrated in Fig. 10, showing the relative performance of each alternative.

**Table 21:** MOORA scores and resulting rankings of dendrimer alternatives based on the weighted evaluation criteria

Rank	Alternative	MOORA Score
1	Phosphorus-containing	0.058866
4	PDI-cored	-0.0803929
3	Aliphatic polyamide	0.0430812
2	Triazine-based	0.0435506

**Table 22:** Normalized decision matrix used in the TOPSIS method, showing scaled values of dendrimer alternatives across all evaluation criteria

Dendrimers	AZI index	F index	NK index	NK$^*$ index
Phosphorus-containing	0.958321	0.982205	0.707107	1.000000
PDI-cored	0.214674	0.170508	0.707107	$9.7613 \times 10^{-35}$
Aliphatic polymide	0.112447	0.059651	$4.9503 \times 10^{-14}$	$3.5148 \times 10^{-68}$
Triazine-based	0.141809	0.035273	$3.0648 \times 10^{-03}$	$3.1064 \times 10^{-44}$

**Table 23:** Weighted normalized decision matrix of dendrimer alternatives, used in the TOPSIS method to calculate relative closeness to the ideal solution

Dendrimers	AZI index	F index	NK index	NK$^*$ index
Phosphorus-containing	0.239580	0.245551	0.176777	0.250000
PDI-cored	0.053668	0.042627	0.176777	$2.4403 \times 10^{-35}$
Aliphatic	0.028112	0.014913	$1.2376 \times 10^{-14}$	$8.7870 \times 10^{-69}$
Triazine-based	0.035452	0.008818	$7.6621 \times 10^{-04}$	$7.7660 \times 10^{-45}$

### TOPSIS method

**Fig. 11:**
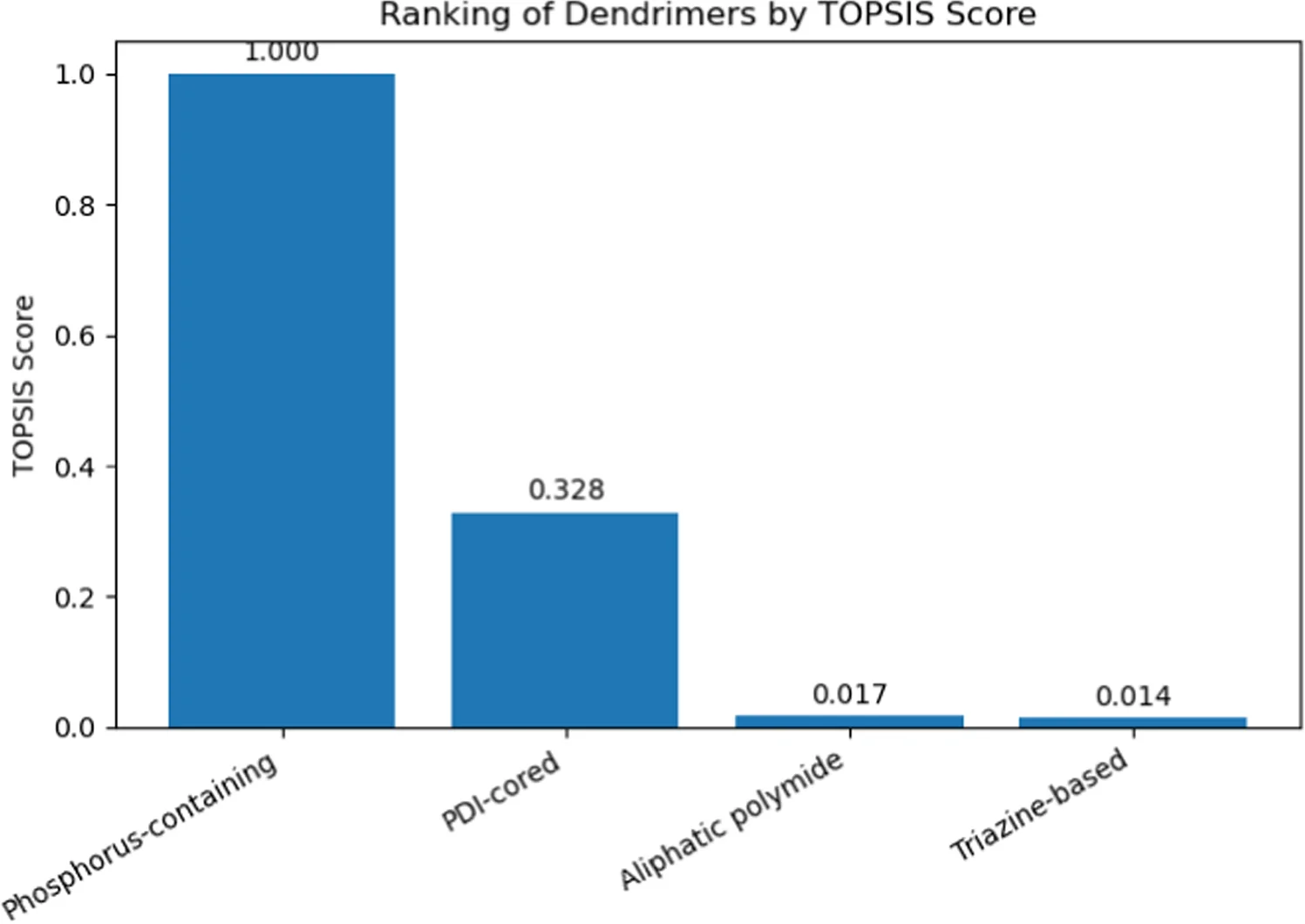
TOPSIS scores and resulting rankings of dendrimer alternatives based on the weighted evaluation criteria

The TOPSIS (Technique for Order of Preference by Similarity to Ideal Solution) method is a widely used multi-criteria decision-making approach for ranking alternatives based on their closeness to an ideal solution [47, 56]. It evaluates each alternative by calculating its distance from a positive ideal solution (best possible values for all criteria) and a negative ideal solution (worst possible values). Alternatives closer to the positive ideal and farther from the negative ideal receive higher preference. This approach ensures that both the optimal and least desirable performance levels are considered simultaneously, providing a balanced and comprehensive ranking. The steps in the TOPSIS method are as follows:

1. * Normalize the decision matrix* This step converts the initial decision matrix 13 into a dimensionless form, allowing all criteria to be compared on a common scale.
  30 $$\begin{aligned} \tilde{R}_{kl} = \frac{R_{kl}}{\sqrt{\sum _{k=1}^{r} R_{kl}^2}} \end{aligned}$$
  Here $R_{kl}$ is the normalized matrix given in Table 22.
2. * Weights of normalized matrix* Calculate the weights of the normalized matrix by using Eq. (31),
  31 $$\begin{aligned} V_{kl} = \tilde{R}_{kl} \, W_{l} \end{aligned}$$
  where $V_{kl}$ denotes the weighted normalized value of the *k*-th alternative with respect to the *l*-th criterion. $V_{kl}$ normalized weights matrix is given in Table 23.
3. *Calculation of ideals* Calculate the ideal best and worst values. Using the above criteria, we determined the ideal best and worst in Table 24. $V_l^{+}$ represents the positive ideal value of the *l*-th criterion, $V_l^{-}$ is negative ideal value and *q* is the total number of criteria considered.
4. *Euclidean distance* Calculate the Euclidean distance from the ideal best
  32 $$\begin{aligned} T^{+}_{k} = \sqrt{\sum _{l=1}^{q} \left( V_{kl} {-} V^{+}_{l} \right) ^{2}}, ~~ T_k^{-} = \sqrt{\sum _{l=1}^{q} \left( V_{kl} {-} V_l^{-} \right) ^2},\nonumber \\ \end{aligned}$$
  where $T_k^{-}$ is the Euclidean distance of the *k*-th alternative from the negative ideal solution and $T_k^{+}$ is the Euclidean distance of the *k*-th alternative from the positive ideal solution.
5. *Performance score* Calculate performance score $S_{k}$ by using Eq. (33)
  33 $$\begin{aligned} S_{k} = \frac{T^{-}_{k}}{T^{+}_{k} + T^{-}_{k}} \end{aligned}$$
  After calculating the performance score, rank the best alternatives according to $S_k$ in descending order. Table 25 shows the performance score and ranking, and is illustrated in Fig. 11, showing the relative performance of each alternative.

**Table 24:** Positive ideal solution ($A^{+}$) and negative ideal solution ($A^{-}$) in the TOPSIS method, representing the best and worst possible criterion values for dendrimer alternatives

	AZI index	F index	NK index	NK$^*$ index
$V_l^{+}$	0.239917	0.245724	0.176776	0.250000
$V_l^{-}$	0.028154	0.008821	0.000000	0.000000

**Table 25:** Final ranking of dendrimer alternatives based on the relative closeness scores ($S_k$) obtained from the TOPSIS method

Rank	Alternative	$S_k$
1	Phosphorus-containing	1.000000
2	PDI-cored	0.328252
3	Aliphatic polymide	0.016595
4	Triazine-based	0.013750

The obtained TOPSIS rankings of dendrimer structures show meaningful correspondence with reported experimental and biomedical findings. Specifically, higher-generation phosphorus-containing dendrimers, which appear closer to an ideal solution in the TOPSIS framework, have been reported to exhibit enhanced drug encapsulation efficiency, improved aqueous solubility, and favorable biocompatibility. These properties are commonly attributed to their dense surface functionality and flexible branching architecture. Similarly, PDI-cored dendrimers, which also rank among the top alternatives in TOPSIS scores in MCDM analysis, have been shown to exhibit strong π- π stacking interactions and enhanced photostability, making them effective candidates for imaging and targeted delivery systems [37]. By contrast, aliphatic dendrimers are usually ranked moderately in line with their low drug loading capability and reduced surface reactivity, as observed in experimental studies [57]. Lastly, the dendrimers that are triazine-based, which are at a relatively lower ranking, have been linked with rigidity and increased cytotoxicity at higher concentration, limiting their biomedical applicability  [1]. These qualitative correspondences suggest that proximity to the ideal solution in the TOPSIS framework reflects a favorable balance of topological and physicochemical descriptors, including branching flexibility, surface reactivity, and molecular polarity. Consequently, the applied MCDM approach provides a chemically meaningful ranking that captures inherent structure–property relationships relevant to dendrimer-based drug delivery. While not a substitute for experimental validation, the proposed framework establishes a predictive baseline that can help guide and prioritize future experimental investigations.

## Stability and sensitivity analysis

To assess the strength and credibility of the suggested MCDM model in different circumstances and uncertainties, a systematic stability and sensitivity analysis was carried out. In particular, three sensitivity analysis have been conducted to investigate the impact of various factors on the final ranking of alternatives.

1. Sensitivity analysis of variation of criteria weights.
2. Sensitivity analysis on Rank Reversal.
3. Sensitivity analysis by various methodologies.

These analyses can be used to provide a very good understanding of the structural stability and reliability of the model proposed, so that the results of the ranking will be sufficiently sensitive to particular assumptions, other alternatives, or methodological options. Indicatively, the sensitivity analysis is considered to be a crucial methodology to evaluate the strength of MCDM results by weight manipulation and variations of alternative sets [58].

### Sensitivity analysis based on variation of criteria weight

To check the consistency of the MCDM criteria, a sensitivity study was conducted following an equal weight approach, wherein each of the four degree-based topological indices (AZI, F, NK, and NK$^*$) was initially assigned an identical weight of 0.25. Since each and every criterion had an equal weight, any of the indices would have been chosen without affecting the original balance. In line with this, the AZI index was selected at random and assigned as a reference criterion within which the variation in weight was to be analyzed. Next, the proportionality formula  [59] was used to compute the elasticity coefficients ($\alpha _\text {c}$) in a manner such that the sum of the weights was one in all different scenarios of the weight variation.

#### Weight elasticity coefficient

The weight elasticity coefficient (EC) expresses how other criteria weights adjust relative to changes in the most influential criterion. For the AZI index as the reference, it is defined as:

34 $$\begin{aligned} \alpha _\text {c} = \frac{w_{{\text {c}}0}}{1 - w_s} \end{aligned}$$

where $w_{{\text {c}}0}$ is the original weight of criterion c and $w_s$ is the original weight of the reference criterion. With $w_{{\text {c}}0} = w_s = 0.25$, the elasticity coefficients were computed as:

35 $$\begin{aligned} \alpha _\text {c} = \frac{0.25}{1 - 0.25} = 0.3333 \end{aligned}$$

#### Determination of Δx

Δx represents the permissible weight change for AZI, maintaining non-negative weights for all criteria:

36 $$\begin{aligned} - w_s \le \Delta x \le \min \left\{ \frac{w_{{\text {c}}0}}{\alpha _\text {c}}\right\} \quad \Rightarrow \quad -0.25 \le \Delta x \le 0.75 \end{aligned}$$

#### Calculation of new weights

Weights for each scenario were updated proportionally as:

37 $$\begin{aligned} w_s&= w_{s0} + \alpha _s \Delta x \end{aligned}$$

38 $$\begin{aligned} w_{\text {c}}&= w_{{\text {c}}0} - \alpha _\text {c} \Delta x \end{aligned}$$

This preserves the condition $\sum w_i = 1$ for all variations. Table 26 summarizes the elasticity coefficients, and Table 27 lists the 22 generated weight scenarios.

**Table 26:** Elasticity coefficients and permissible weight variation ranges for the topological indices

Criteria	Weight ($w_0$)	EC ($\alpha _\text {c}$)	Δx Range
AZI index (most important)	0.25	1.0000	-0.25→+0.75
F index	0.25	0.3333	up to 0.75
NK index	0.25	0.3333	up to 0.75
NK$^*$ index	0.25	0.3333	up to 0.75

The calculated coefficients were then used to obtain new weights for each value of Δx across the allowed range, producing 22 scenarios.

**Table 27:** New criteria weights across 22 sensitivity scenarios under varying Δx

Scenario	$\boldsymbol{\Delta x}$	AZI ($w_s$)	F ($w_{\text {c}}$)	NK ($w_{\text {c}}$)	NK$^*$($w_{\text {c}}$)	Total
S1	-0.250	0.0000	0.3333	0.3333	0.3333	1.000
S2	-0.202	0.0476	0.3175	0.3175	0.3175	1.000
S3	-0.155	0.0952	0.3016	0.3016	0.3016	1.000
S4	-0.107	0.1429	0.2857	0.2857	0.2857	1.000
S5	-0.060	0.1905	0.2698	0.2698	0.2698	1.000
S6	-0.012	0.2381	0.2540	0.2540	0.2540	1.000
S7	0.036	0.2857	0.2381	0.2381	0.2381	1.000
S8	0.083	0.3333	0.2222	0.2222	0.2222	1.000
S9	0.131	0.3810	0.2063	0.2063	0.2063	1.000
S10	0.179	0.4286	0.1905	0.1905	0.1905	1.000
S11	0.226	0.4762	0.1746	0.1746	0.1746	1.000
S12	0.274	0.5238	0.1587	0.1587	0.1587	1.000
S13	0.321	0.5714	0.1429	0.1429	0.1429	1.000
S14	0.369	0.6190	0.1270	0.1270	0.1270	1.000
S15	0.417	0.6667	0.1111	0.1111	0.1111	1.000
S16	0.464	0.7143	0.0952	0.0952	0.0952	1.000
S17	0.512	0.7619	0.0794	0.0794	0.0794	1.000
S18	0.560	0.8095	0.0635	0.0635	0.0635	1.000
S19	0.607	0.8571	0.0476	0.0476	0.0476	1.000
S20	0.655	0.9048	0.0317	0.0317	0.0317	1.000
S21	0.702	0.9524	0.0159	0.0159	0.0159	1.000
S22	0.750	1.0000	0.0000	0.0000	0.0000	1.000

**Fig. 12:**
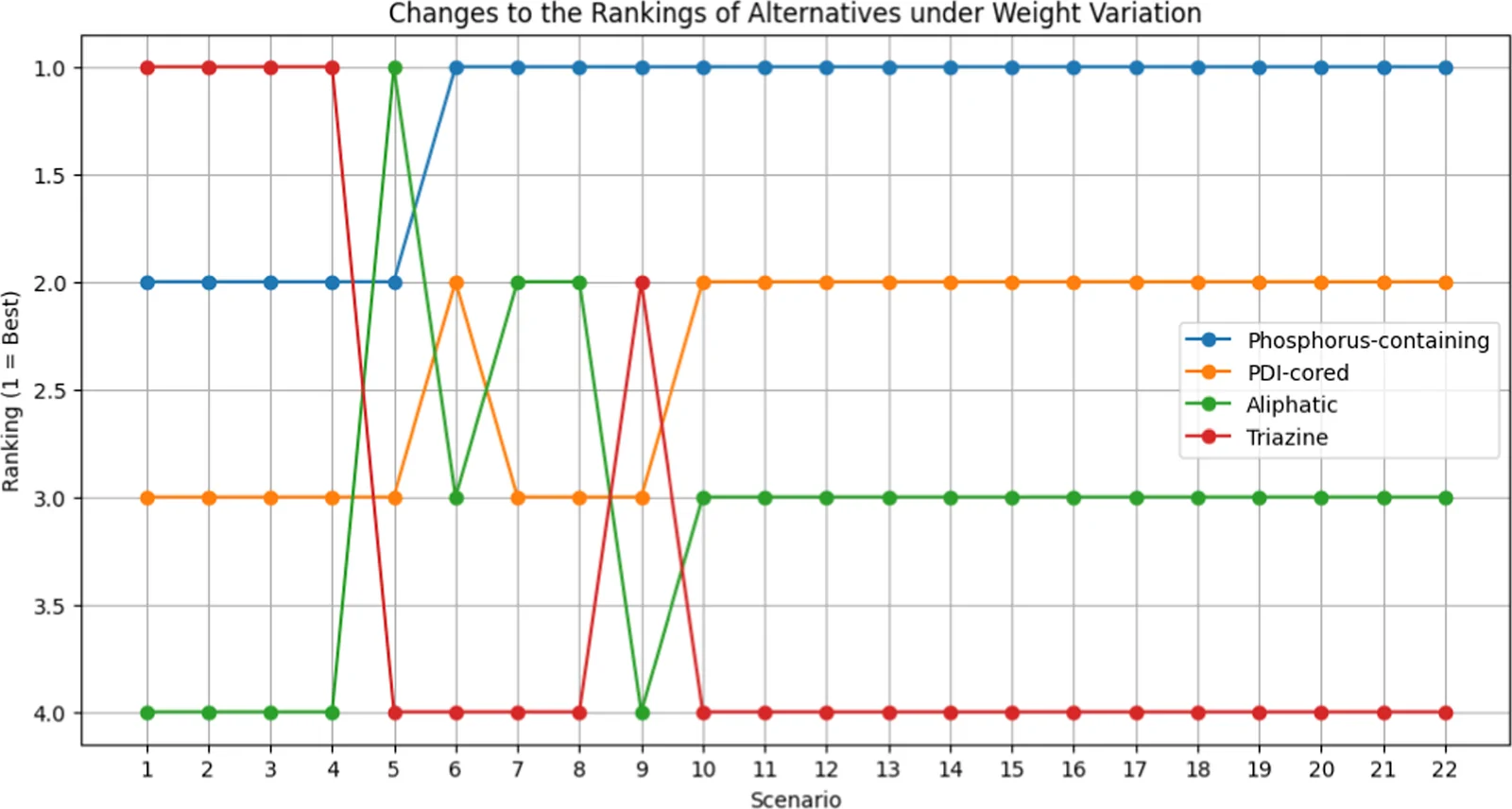
Rankings of alternatives across sensitivity scenarios with varying criterion weights

As Δx increases, AZI becomes dominant, reaching w=1.0 at Δx=0.75, while other criteria decline to zero. Conversely, at Δx=-0.25, AZI weight drops to zero, and the remaining criteria share the total weight equally. Re-calculated rankings of alternatives in all scenarios (Fig. 12 indicate that the high-ranking option, phosphorus-containing dendrimer, is consistent under realistic changes in the weight of AZI, and the fluctuations in the rankings of other options are only apparent with the application of extreme weight reductions of AZI. This shows that the model recognizes some critical areas of sensitivity, and the decision-making process remains consistent and transparent. The sensitivity analysis, cumulatively, indicates that the model is robust, consistent, and capable of identifying critical sensitivity points and, at the same time, remaining reliable in making decisions.

**Fig. 13:**
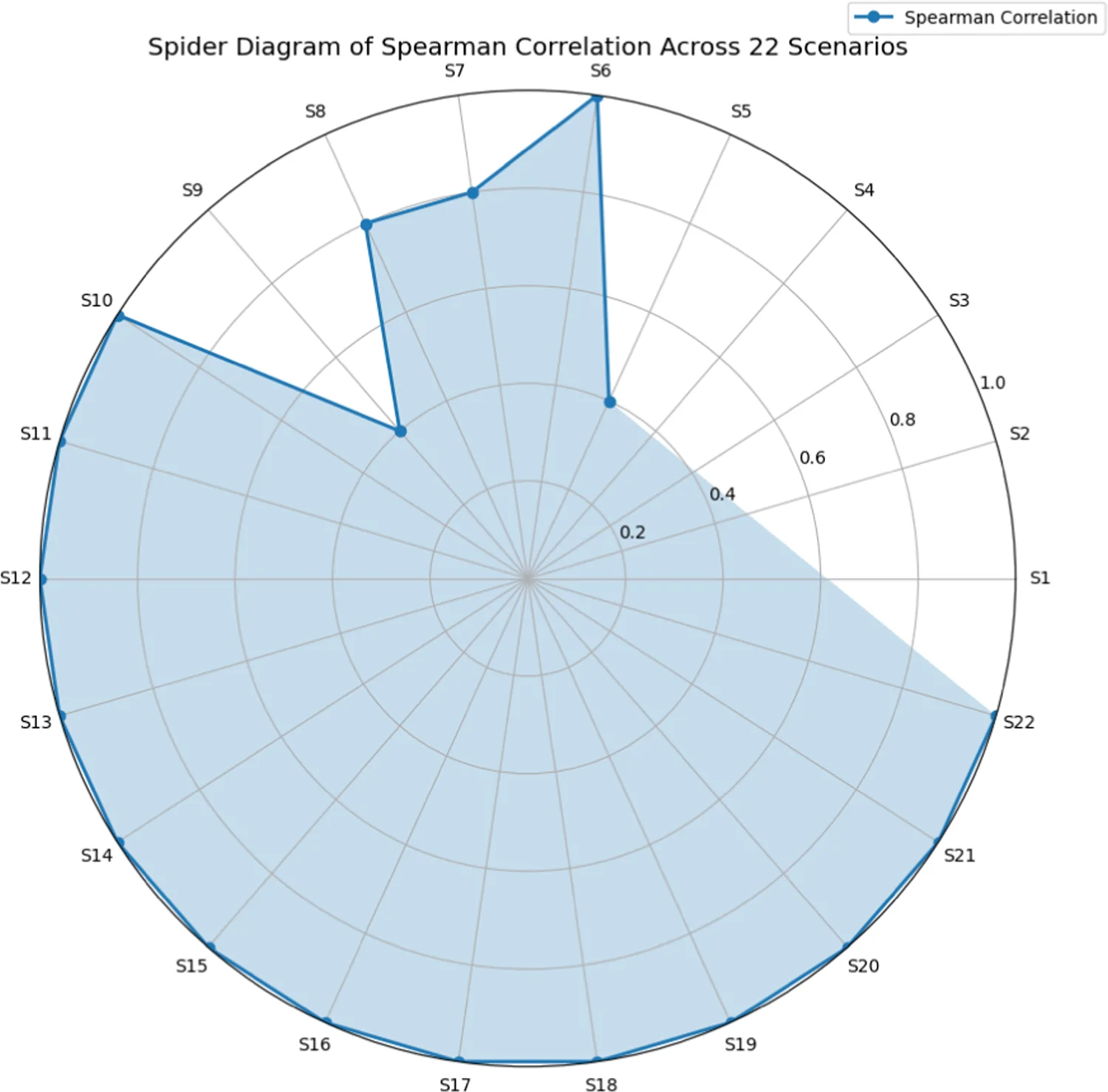
Spider diagram illustrating the Spearman rank correlation coefficients between the original ranking (scenario S10) and rankings under 22 different weight variation scenarios. Each axis represents a scenario, and the radial distance indicates the correlation strength, ranging from 0 (no correlation) to 1 (perfect correlation)

The Spearman rank correlation coefficients were determined using the original ranking (scenario S10) as the basis of comparison against the rankings of 22 scenarios of varying weights, as illustrated in Fig. 13, these coefficients are statistically significant, demonstrating that although ranking fluctuations occur under extreme weight changes, the model overall maintains consistent and robust performance across the majority of weight variations.

#### Sensitivity analysis based on rank reversal

One way to assess the stability of the MCDM methods is to add new alternatives to the initial set of alternatives or delete weaker alternatives. Under these circumstances, an effective MCDM approach will retain the ranking of the initial alternatives relatively with minimal variations. This is referred to as the rank reversal problem and has been a considerable topic in the literature [60]. In order to determine the reliability of the obtained results provided by the proposed model, dynamic decision matrices were developed by adding or removing the alternatives systematically. The rankings were calculated in these altered conditions, and the generated rankings were compared with the original rankings. In case the rankings of the top options are relatively stable, the model can be assumed to be stable and resistant to rank reversal, and there is confidence in the use of the model in decision-making applications.

In order to test the robustness of the suggested MCDM framework in terms of rank reversal results, four scenarios (S0–S3) were designed. In the successive scenarios, the dendrimer of the lower rank in the previous scenario was eliminated, and the rest of the alternatives were reconsidered with the help of the SAW method. The weighted scores were obtained by normalizing the decision matrix Table 18 and assigning weights to the criteria.

Table 28 shows a summary of the SAW score and ranking of dendrimers in these four scenarios. A phosphorus-containing dendrimer is always ranked in the first position with a high score, demonstrating its clear dominance irrespective of the elimination of the other options. Moreover, the rankings and scores of the remaining options would not change as the dendrimers are sequentially eliminated, meaning that the system of rankings is very robust to alterations in the set of options.

**Fig. 14:**
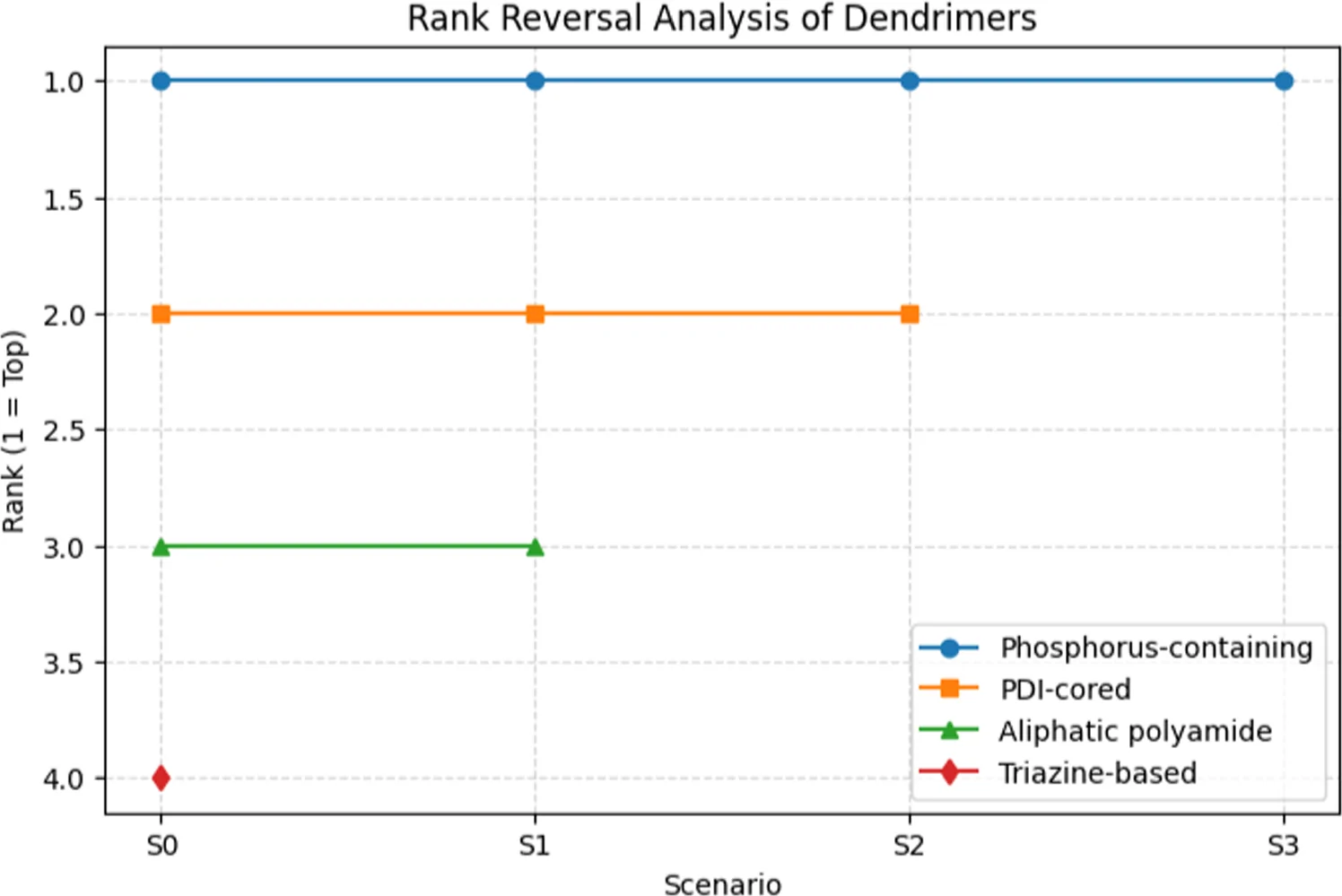
Results of Rank Reversal Analysis

Analysis of rank reversal (Fig. 14) demonstrates that the phosphorus dendrimer is always ranked highest in all the situations, and lower-ranked dendrimers do not change the top positions, which proves the validity and robustness of the framework.

#### Sensitivity analysis of ranking stability based on different ranking methodologies

**Fig. 15:**
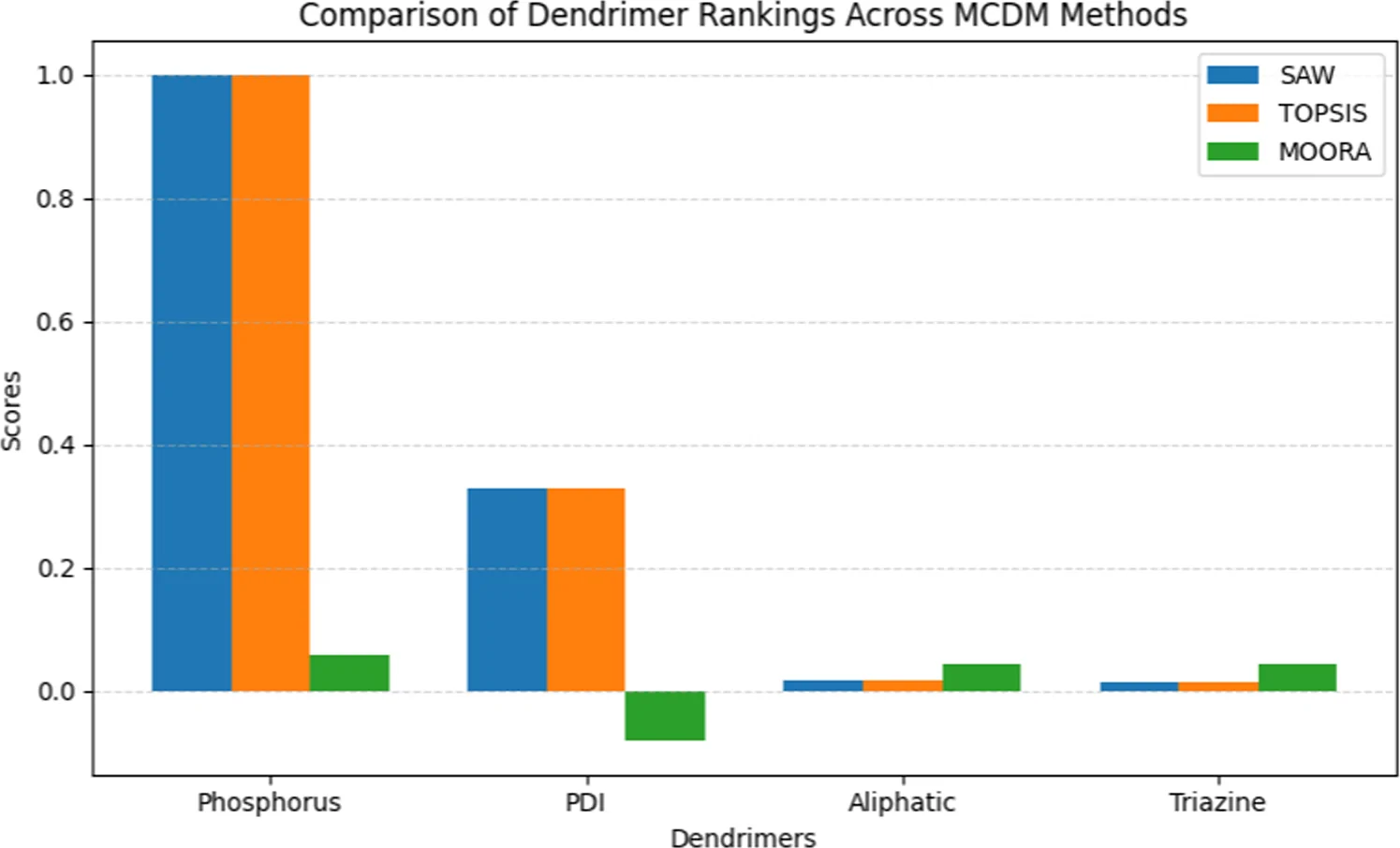
Comparative ranking of all considered dendrimers across multiple MCDM methods

The dendrimer rankings were compared between three well-established and reliable methods, namely: SAW, TOPSIS, and MOORA, to evaluate the robustness and reliability of the proposed MCDM model [59]. These cross-method comparisons help to identify the consistency of prioritization of alternatives when it is not based on one framework. As has been analyzed, the best-performing dendrimers, and especially the phosphorus-containing and the PDI-cored, consistently maintained their top positions in all methods and situations. This confirms the stability and reliability of the proposed framework. Figure 15 shows the comparative rankings of all dendrimers as a graphical representation of how the various methods agree on the rankings of all dendrimers.

**Table 28:** Ranking scores of dendrimers under different removal scenarios

Dendrimer	S0: All 4	S1: Remove triazine	S2: Remove aliphatic	S3: Remove PDI
Phosphorus-containing	0.8035 (1)	0.8035 (1)	0.8035 (1)	0.8035 (1)
PDI-cored	0.1563 (2)	0.1563 (2)	0.1563 (2)	—
Aliphatic polyamide	0.0204 (3)	0.0204 (3)	—	—
Triazine-based	0.0193 (4)	—	—	—

In Table 29, Spearman Rank Correlation (SRC) analysis indicates a correlation between the rankings that are generated by the three MCDM methods, SAW, TOPSIS, and MOORA. This indicates that there is no difference in ranking orders of the dendrimers with all methods, and thus it proves that there is complete agreement and consistency among the various decision-making methods. The process of making a thorough decision that is effective also requires a deep sensitivity analysis, including variations of the criteria weight and reversion of ranks, as well as multiple methodologies. The overall outcomes of these analyses prove that the suggested framework is reliable, consistent, and robust. The dendrimer rankings stay consistent among various MCDM techniques, weight variation, and elimination of alternatives, which provides a reliable basis for the use of dendrimers in drug delivery efforts.

From a chemical perspective, the observed convergence of rankings for phosphorus-containing and PDI-cored dendrimers across different MCDM methods and sensitivity scenarios indicates that their underlying structural characteristics exert a dominant and consistent influence on the selected evaluation criteria. In particular, features such as high branching efficiency, dense and uniform surface functionality, and balanced molecular connectivity contribute to favorable performance across multiple criteria simultaneously, making these dendrimer architectures less sensitive to variations in weighting schemes. In contrast, dendrimer systems that do not consistently retain their rank under sensitivity analysis are more affected by structural trade-offs, such as increased rigidity, lower surface reactivity, or uneven branching patterns. These trade-offs amplify the influence of individual criteria when weights are varied, leading to minor ranking fluctuations. Thus, ranking convergence reflects chemically robust and well-balanced dendrimer architectures, whereas limited divergence highlights sensitivity to specific structural features relevant to drug-delivery performance.

**Table 29:** Spearman rank correlation (SRC) between SAW, TOPSIS, and MOORA rankings of dendrimers

	SAW	TOPSIS	MOORA
SAW	1.00	1.00	1.00
TOPSIS	1.00	1.00	1.00
MOORA	1.00	1.00	1.00

## Linear modeling of properties of dendrimer generation

Regression analysis serves as a means to explore how various properties are associated with distance–based topological descriptors. A common approach within this framework is the method of least squares, which is employed to quantify relationships between variables. The simplest and most widely used variant of linear regression is the linear least squares fitting technique, where one determines the straight line that best represents a given set of data points. After computing correlation coefficients for each index with respect to the properties of interest, the index exhibiting the strongest correlation is selected as the most suitable descriptor [61]. A correlation coefficient takes values in the interval -1 to 1 and reflects both the magnitude and direction of correlation between two variables [62]. A value close to +1 or -1 indicates an excellent fit.

**Fig. 16:**
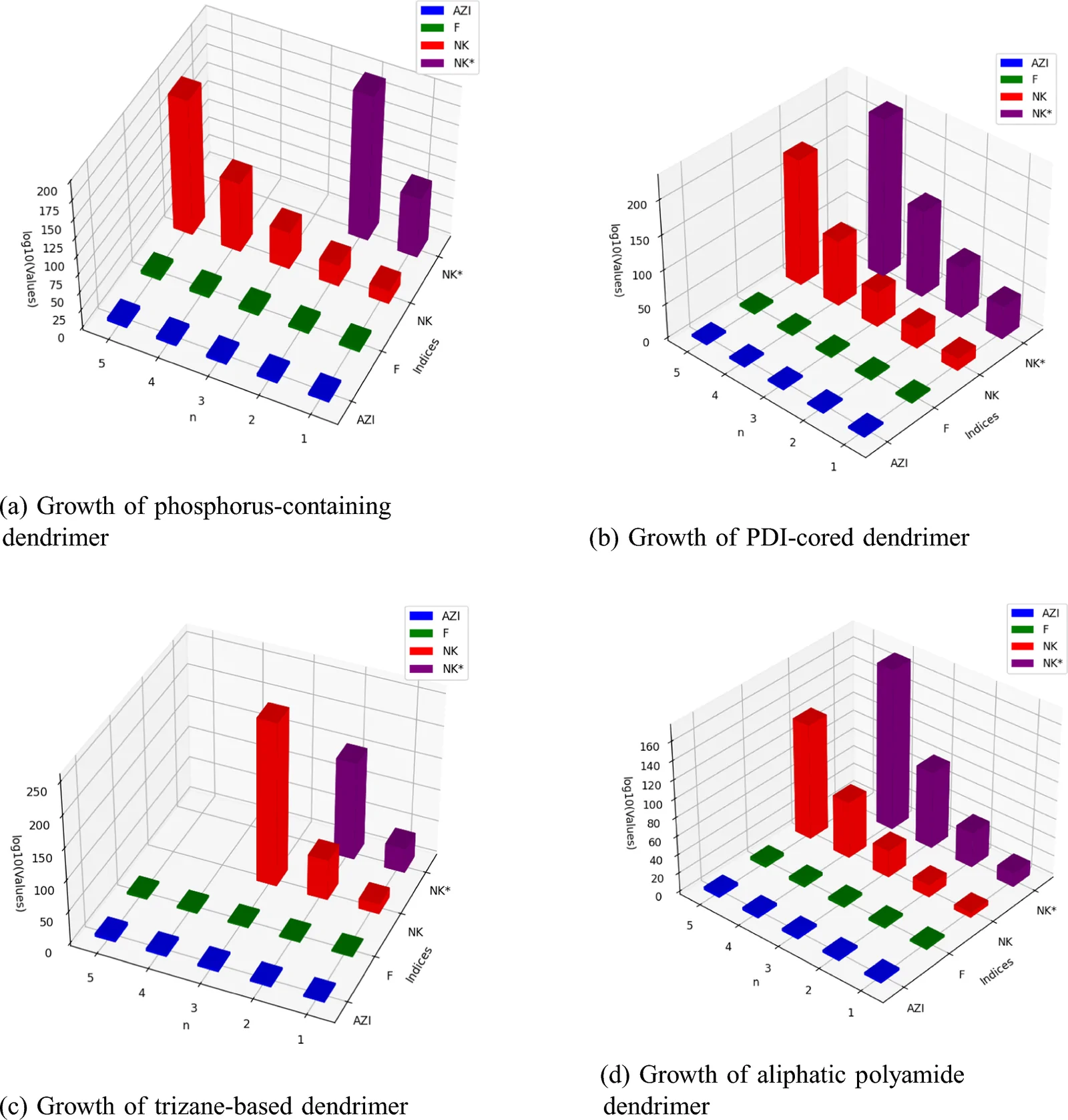
Growth of the considered dendrimers up to the fifth generation: (a) Growth of phosphorus-containing dendrimer, (b) Growth of PDI-cored dendrimer, (c) Growth of triazine-based dendrimer, and (d) Growth of aliphatic polyamide dendrimer

**Fig. 17:**
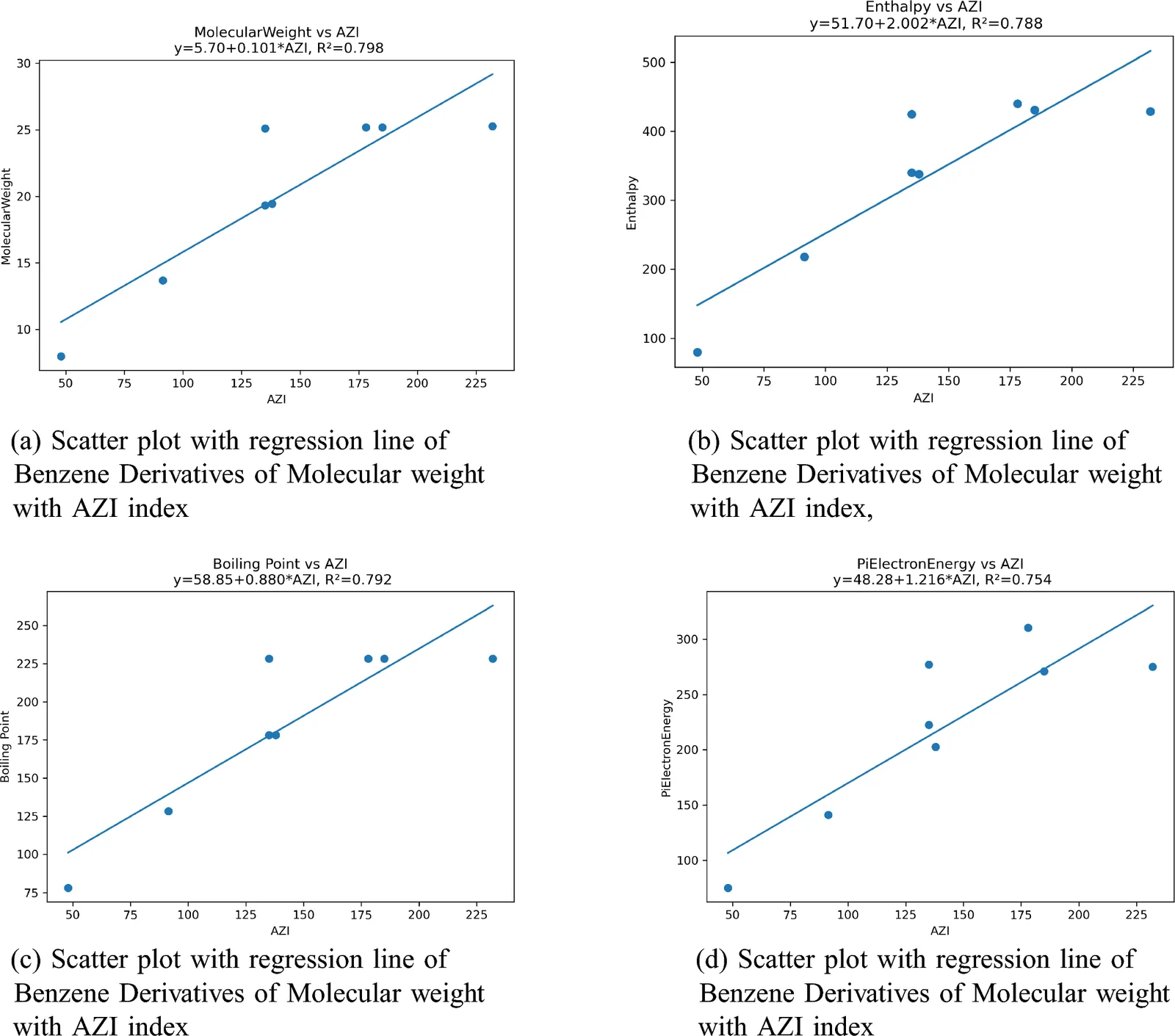
Scatter plots with regression lines showing the correlation between the Augmented Zagreb Index (AZI) and physicochemical properties of representative benzene derivatives used as a reference dataset for descriptor evaluation in the dendrimer ranking framework: (a) molecular weight vs. AZI index, (b) enthalpy vs. AZI index, (c) boiling point vs. AZI index, and (d) π-electron energy vs. AZI index

A simple linear regression model was used to evaluate the predictive ability of the topological indices using each of the topological indices versus some of the physicochemical properties, which were molecular weight, enthalpy, boiling point, and π-electron energy. To quantify the relationship between topological descriptors and the selected physicochemical properties, a simple linear regression model of the form

39 $$\begin{aligned} Y = \alpha + \beta X + \varepsilon \end{aligned}$$

was employed, where *Y* denotes the target molecular property, *X* represents the corresponding degree-based topological index, α and β are regression coefficients, and ε is the error term. To measure the strength of correlation, regression equations and regression coefficients of determination were computed. Figure 17 displays representative regression plots with $R^2$ values ranging from 0.75 to 0.82, suggesting that AZI predicts experimental properties well; the molecular complexity defined by AZI is high enough to indicate changes in size and atomic composition of the derivatives (Fig. 17a). On the same note, enthalpy was found to have a strong correlation with AZI, indicating that AZI captures structural information related to thermodynamic stability (Fig. 17b). The fact that AZI is sensitive to the intermolecular interactions and structural branching (boiling point correlation, $R^2 = 0.792$) further demonstrates that the index is sensitive to the phenomena affecting the behavior of the phase transition (Fig. 17c). Moreover, the correlation between the energy of the π-electrons and that of AZI asserts the capacity of the said topological index in defining the electronic characteristics that relate to the aromaticity and conjugation (Fig. 17d). The regression analysis is intended to provide a first-order assessment of the predictive relevance of degree-based topological indices rather than a fully optimized QSPR model. The observed correlations indicate systematic trends between molecular structure and properties, supporting the use of these indices as informative predictors within the proposed decision-making framework.

Overall, the results demonstrate that AZI serves as a reliable topological descriptor, capturing both physicochemical and electronic characteristics of benzene derivatives. The relatively high $R^2$ values across all properties confirm its predictive potential in structure–property relationship modeling. Figure 16 illustrates the growth of dendrimers across generations. Notably, the NK and NK$^{*}$ indices exhibit exponential growth with increasing dendrimer generation. This behavior arises because NK-type indices are degree-based descriptors that amplify contributions from vertices with higher connectivity. As dendrimers expand radially from generation n=1 to n=7, the number of branching sites increases geometrically, leading to an exponential increase in vertex degrees as in Tables 30, 31, 32 and 33. The observed exponential trend in the NK and NK$^{*}$ index reflects the rapid increase in vertex degrees associated with successive dendrimer generations. Chemically, this behavior arises from the repetitive and hierarchical branching mechanism inherent to dendrimer growth, where each new generation multiplies the number of terminal and internal connectivity points. As a result, NK and NK$^{*}$ capture the accelerating expansion of local bonding environments, which correlates with increased molecular complexity and surface functionality. This exponential behavior highlights the sensitivity of degree-based indices to dendrimer generation growth and underscores their relevance in modeling structural scalability and drug-loading potential.

**Table 30:** Growth pattern of the phosphorus-containing dendrimer, illustrating the expansion of generations and branching structure

*n*	AZI	F	NK	NK$^*$
1	2578.97	5070	$4.738381338 \times 10^{18}$	$6.496426103 \times 10^{82}$
2	5755.3562	11142	$5.215788145 \times 10^{29}$	$4.3383411258 \times 10^{196}$
3	12108.1286	23286	$6.319748715 \times 10^{51}$	$1.934376719 \times 10^{424}$
4	24813.6734	47574	$9.278110596 \times 10^{95}$	$3.847856511 \times 10^{879}$
5	50224.763	96150	$1.999765237 \times 10^{184}$	$1.5219923092 \times 10^{1790}$
6	101046.9422	193302	$2.900479094 \times 10^{360}$	$2.3812021645 \times 10^{3611}$
7	202691.3006	387606	$2.004914385 \times 10^{714}$	$5.82872806925 \times 10^{7253}$

**Table 31:** Growth pattern of the PDI-cored dendrimer, illustrating the expansion of generations and branching structure

*n*	AZI	F	NK	NK$^*$
1	577.59375	880	$4.738381338 \times 10^{18}$	$6.341180737 \times 10^{48}$
2	883.09375	1316	$5.215788145 \times 10^{29}$	$5.041038762 \times 10^{74}$
3	1494.09375	2188	$6.319748715 \times 10^{51}$	$3.185089238 \times 10^{126}$
4	2716.09375	3932	$9.278110596 \times 10^{95}$	$1.272837716 \times 10^{230}$
5	5160.09375	7420	$1.999765237 \times 10^{184}$	$2.029638769 \times 10^{437}$
6	10048.09375	14396	$2.900479094 \times 10^{360}$	$5.164368115 \times 10^{851}$
7	19824.09375	28348	$2.004914385 \times 10^{714}$	$3.3453987615 \times 10^{1680}$

**Table 32:** Growth pattern of the triazine-based dendrimer, showing its generational expansion and branching structure

*n*	AZI	F	NK	NK$^*$
1	381.5625	182	$2.054269543 \times 10^{16}$	$2.0184240983 \times 10^{39}$
2	1779.8125	494	$1.9787330143 \times 10^{64}$	$2.2767856975 \times 10^{154}$
3	7372.8125	1118	$1.703389393 \times 10^{256}$	$3.6860483793 \times 10^{614}$
4	29744.8125	2368	$9.353860846 \times 10^{1023}$	$2.53230977518 \times 10^{2455}$
5	119232.8125	4862	$8.50528975335 \times 10^{4094}$	$5.64073852354 \times 10^{9818}$
6	477184.8125	9584	$5.81446824299 \times 10^{16378}$	$1.38377426938 \times 10^{39272}$
7	1909892.813	19833	$1.26998013852 \times 10^{65154}$	$5.10269921163 \times 10^{157085}$

**Table 33:** Growth pattern of the aliphatic polyamide dendrimer, showing its generational expansion and branching structure

*n*	AZI	F	NK	NK$^*$
1	302.6349728	308	331776	$2.28325217148 \times 10^{15}$
2	540.9124556	780	$2.8179280429 \times 10^{13}$	$8.7407707872 \times 10^{37}$
3	1017.467421	1724	$2.0328239245 \times 10^{29}$	$1.2817973204 \times 10^{83}$
4	1970.577352	3612	$1.0578875156 \times 10^{61}$	$2.7565039212 \times 10^{173}$
5	3876.797215	7388	$2.8649625491 \times 10^{124}$	$1.2747855299 \times 10^{354}$
6	7689.23964	14940	$2.10125066843 \times 10^{251}$	$2.7264287095 \times 10^{715}$
7	15314.11639	30044	$1.1303051147 \times 10^{505}$	$1.2471198404 \times 10^{1433}$

## Discussion

The structural interpretations of the selected topological descriptors, introduced in Sect. 3, provide a useful basis for understanding the computational results and their implications for dendrimer-mediated drug delivery. The obtained rankings and descriptor-property relationships are also consistent with several previously reported experimental and biomedical studies. In particular, experimentally, phosphorus-based higher generations of dendrimers have been shown to have higher drug encapsulation, greater biocompatibility, and better aqueous solubility because of their dense surface functionality and their flexible branching structure [63, 64]. Likewise, PDI-cored dendrimers that are also among the best alternatives in our MCDM analysis have been demonstrated to be of excellent quality in terms of being capable of having high π- π stacking interactions and enhanced photostability and are, therefore, living up to being potential candidates in imaging and targeted delivery systems  [37]. Contrastingly, aliphatic dendrimers tend to be ranked in the middle range, in line with the relatively low drug loading capacity and reduced surface reactivity, as was also found in experimental research studies [57]. Lastly, the triazine-based dendrimers, which are ranked comparatively lower, have been linked with enhanced rigidity and amplified cytotoxicity at higher concentrations, limiting their usability in biomedical applications  [38]. These qualitative correspondences reveal that the topological and physicochemical descriptors used here reflect intrinsic properties of the molecules, such as surface reactivity, polarity, and branching flexibility, that have a profound connection to the experimentally observable properties, such as encapsulation efficiency, biocompatibility, and stability. Both phosphorus-containing and PDI-core dendrimers are placed in the first positions of the list of the theoretical rankings, which correlate well with the experimental literature reportage of higher drug-loading capability and sustained release behavior  [65]. These similarities demonstrate that the proposed computational framework is useful for modeling important structural and physicochemical determinants of the real-world performance of drug delivery. Therefore, the theoretical framework in this paper offers a predictive ground that is consistent and can actually inform future investigations through experiments.

## Conclusion

This paper shows a theoretical framework that provides a detailed analysis of the structural and physicochemical properties of the dendrimers by using topological and decision-making techniques. To measure different facets of molecular connectivity and branching complexity, the AZI, F-index, Narumi Katayama index, as well as the Modified Narumi- Katayama index were used. Among these, the F-index is effective at presenting the role of branching on reactivity and stability of molecules, and the AZI offers a more comprehensive view of atomic connectivity by combining both vertex and edge interactions. A combination of these indices provides a comprehensive description of the dendrimer topology and is used as a basis for the multi-criteria assessment.

Four families of dendrimers, phosphorus, PDI, triazine, and aliphatic polyimide, based on their topological descriptors and physicochemical properties, were comparatively ranked using the MCDM analysis. The comparison has shown that the phosphorus and PDI-based dendrimers have better overall operation characteristics that are provided by higher compactness of the structure and thermodynamic stability, and trizaine and aliphatic polyamide dendrimers exhibit moderate performance characteristics, which are shown by a low degree of branching and connectivity. These tendencies are in line with reported experimental results indicating chemical versatility and high drug loading capability of phosphorus-containing dendrimers.

Although these results are quite promising, the current work has limitations, such as being theoretical because the graph-based models simplify the real molecular geometry, solvent interactions, and biological responses. In addition, the results of the MCDM are dependent on the selected physicochemical parameters and the weights that can be different in various experimental conditions. Future studies, however, ought to be directed to confirm these computational rankings by means of experimental assays such as cytotoxicity, encapsulation challenge, and kinetic release studies, in addition to quantum-precise chemical simulations and molecular dynamics simulations. When topological indices and decision analysis techniques are used together, this creates a predictive and consistent framework for the rational design of dendrimers. The strategy offers useful information about structure–property relationships, as well as a good theoretical foundation for optimization of the scaffold structures of the dendrimers in drug delivery, nanomedicine, and other applications. Although this is a computational study, the predictions are made using theoretical models that have been calibrated using available experimental data and can be used by chemists or pharmaceutical researchers to experimentally justify such predictions.

## Data Availability

The data are available upon reasonable request to the corresponding authors.
